# Maternal autoimmune diseases during pregnancy and mortality up to young adulthood in offspring: a register-based cohort study

**DOI:** 10.1016/j.eclinm.2026.104237

**Published:** 2026-09-22

**Authors:** Huan Zhang, Imre Janszky, Elizabeth V. Arkema, Jette Möller, Yajun Liang, Xingbo Mo, Krisztina D. László

**Affiliations:** a MOE Key Laboratory of Geriatric Diseases and Immunology, Department of Epidemiology, School of Public Health, Suzhou Medical College of Soochow University, Suzhou, China; b Department of Global Public Health, Karolinska Institutet, Stockholm, Sweden; c Department of Public Health and Nursing, Norwegian University of Science and Technology, Trondheim, Norway; d Division of Clinical Epidemiology, Department of Medicine Solna, Karolinska Institutet, Stockholm, Sweden; e Department of Public Health and Caring Sciences, Uppsala University, Uppsala, Sweden

**Keywords:** Maternal autoimmune disease, Early-life mortality, Perinatal factors, Offspring, Life-course epidemiology

## Abstract

**Background:**

The implications of maternal autoimmune diseases for offspring survival after infancy remain poorly understood. We aimed to examine the association between maternal autoimmune diseases during pregnancy and all-cause and cause-specific mortality in offspring from birth to early adulthood.

**Methods:**

In this nationwide population-based cohort study, we included all live births in Sweden between 2001 and 2014 and followed individuals until death, emigration, or December 31, 2023. Cox proportional hazards models were used to analyze the association between maternal autoimmune diseases during pregnancy and risk of mortality in offspring. To control for familial factors, we conducted cousin and paternal negative control analyses. Mediation analyses were performed to quantify the contribution of adverse perinatal outcomes.

**Findings:**

Among 1,455,645 offspring followed for a median of 15.3 years (interquartile range 11.9–19.0), maternal autoimmune diseases were associated with an increased risk of all-cause mortality in offspring (HR 1.22, 95% CI 1.10–1.36). Associations were evident during infancy and attenuated with higher age. No clear association was observed for paternal autoimmune diseases, and cousin analyses yielded attenuated but directionally consistent estimates. Preterm birth and small for gestational age explained a proportion of the observed associations.

**Interpretation:**

Maternal autoimmune diseases are associated with increased offspring mortality risk, with the excess risk primarily concentrated in early life and driven by deaths related to perinatal conditions and congenital malformations. These findings are consistent with a contribution of intrauterine mechanisms to the observed associations, although other maternal pathways may also contribute.

**Funding:**

The study was supported by grants from the Swedish Heart and Lung Foundation (20230493, 20241167), Karolinska Institutet Research Foundation (2024-02588) and the 10.13039/501100006636Swedish Research Council for Health, Working Life and Welfare (2015-00837).

**Research in context**

**Evidence before this study**

Previous studies have provided evidence of associations between maternal autoimmune diseases and offspring health, particularly for adverse pregnancy outcomes. However, this evidence has largely been derived from studies of individual autoimmune conditions, most commonly type 1 diabetes and systemic lupus erythematosus, or has focused on short-term outcomes such as perinatal complications. Evidence on offspring mortality beyond infancy is scarce, and few population-based studies have examined cause-specific mortality across a broad spectrum of maternal autoimmune diseases.

**Added value of this study**

In this nationwide cohort of more than 1.4 million individuals with up to two decades of follow-up, we provided a broad assessment of maternal autoimmune diseases in relation to offspring mortality. We showed that maternal autoimmune diseases are associated with an increased risk of offspring death, but that this excess risk was not uniform across maternal autoimmune diseases, causes of death or mortality across the life course. Instead, it was primarily driven by deaths from conditions originating in the perinatal period and congenital anomalies, and was evident during infancy. When integrating paternal negative control analyses and family-based comparisons, our findings were consistent with the hypothesis that shared familial factors alone may not fully explain the observed associations and support a possible contribution of intrauterine mechanisms. Mediation analyses further suggested that adverse perinatal outcomes may contribute to the observed association, although the magnitude of mediation was estimated with considerable uncertainty. Taken together, our findings suggest that the association between maternal autoimmune diseases and offspring mortality is developmentally timed, cause-specific, and may partly reflect early-life biological mechanisms.

**Implications of all the available evidence**

The available evidence suggested that maternal autoimmune diseases were associated with offspring survival predominantly through pathways operating during fetal development and the perinatal period, thereby concentrating excess risk to early life rather than later disease processes. This pointed to maternal immune health as a potential factor of early-life vulnerability and to pregnancy and the perinatal period as critical windows for intervention. If our findings are confirmed by future studies, optimizing maternal disease control before and during pregnancy, alongside improved perinatal care, may offer opportunities to reduce preventable early-life mortality.

## Introduction

Mortality in early life remains a major global health challenge, reflecting profound inequalities across populations and health systems.^1^, ^2^, ^3^, ^4^ A large proportion of these deaths are linked to conditions arising before or around birth, underscoring the importance of identifying early-life determinants of long-term survival.^5^, ^6^, ^7^ Increasing attention has therefore been directed toward the role of maternal health during pregnancy in shaping offspring survival across the life course.^8^, ^9^, ^10^ In particular, maternal immune dysregulation has emerged as a potential determinant of offspring health, given its capacity to influence placental function, fetal development, and immune programming.^11^, ^12^, ^13^ Autoimmune diseases, which affect a substantial proportion of women of reproductive age,^14^^,^^15^ are characterized by chronic inflammation and aberrant immune responses, and have been consistently associated with adverse pregnancy outcomes, including preterm birth and abnormal fetal growth,^16^, ^17^, ^18^, ^19^, ^20^, ^21^, ^22^, ^23^ which may in turn lead to offspring death.^24^, ^25^, ^26^, ^27^, ^28^, ^29^ However, whether maternal autoimmune diseases translate into increased mortality risk in offspring beyond the perinatal period remains unclear.

Empirical evidence on the long-term consequences of maternal autoimmune diseases for offspring survival is limited and fragmented. Prior studies have largely focused on individual maternal conditions—most notably maternal type 1 diabetes,^30^, ^31^, ^32^ systemic lupus erythematosus,^33^ or celiac disease^34^—or on short-term outcomes such as perinatal mortality and early-life morbidity. Findings from these studies have been inconsistent, with some suggesting increased mortality in exposed offspring, while others reporting little or no association.^35^ Importantly, most investigations have been limited by modest sample sizes, short follow-up, or a narrow focus on specific diseases or early-life endpoints. Few large, population-based studies have examined multiple maternal autoimmune diseases and offspring all-cause and cause-specific mortality across childhood and early adulthood. Whether these associations reflect intrauterine effects or shared familial factors, and are mediated through adverse birth outcomes and offspring disease remains unclear.

To fill these gaps, we conducted a nationwide cohort study of over 1.4 million Swedish births, followed from birth into young adulthood. We examined the associations of maternal autoimmune diseases with offspring all-cause and cause-specific mortality, leveraging family-based designs to account for shared genetic and environmental influences, and further explored potential mediation through perinatal factors.

## Methods

### Study design, setting and population

We constructed a nationwide birth cohort by linking individual-level data from six Swedish health and socioeconomic registers, i.e., the Medical Birth Register (MBR), the Total Population Register, the Multi-Generation Register, the Register of Education, the Patient Register and the Cause of Death Register. These registers have high coverage and validity.^36^, ^37^, ^38^, ^39^ Record linkage at individual level was possible through the unique personal identity number assigned to all residents in Sweden. The study included all live-born singletons recorded in the MBR between January 1, 2001, and December 31, 2014 (n = 1,455,645). We followed study participants from birth and until death, emigration (available until December 31, 2014), or December 31, 2023, whichever occurred earliest.

### Ethics

Ethical approval for the study was granted by the Research Ethics Committee at Karolinska Institutet, Stockholm (No. 2016/288-31/1, 2021-03315 and 2025-04808-02). The reporting of this observational study was guided by the Strengthening the Reporting of Observational Studies in Epidemiology (STROBE) guidelines. The requirement for individual informed consent was waived because the study used routinely collected register-based data.

### Study variables and their sources

#### Autoimmune diseases

Exposure to maternal autoimmune diseases during pregnancy was defined as any autoimmune disease diagnosis recorded before or during the index pregnancy, based on information from the Patient Register and the MBR. The Patient Register captures both primary and secondary diagnoses from inpatient (available since 1964, nationwide since 1987) and specialized non-private outpatient care (nationwide since 2001). Paternal autoimmune conditions were identified similarly from the Patient Register. Analyses considered both a composite measure of any autoimmune disease and specific disease categories. Autoimmune disorders were grouped into nine predefined categories—endocrine, inflammatory arthritis, vasculitis, connective tissue, skin, blood, nervous system, gastrointestinal, and other autoimmune diseases—following the framework established by Song et al.^40^ and using the International Classification of Diseases (ICD) codes presented in Supplementary Table S1. Validation work on the Patient Register has found generally high diagnostic validity for several autoimmune conditions recorded in the Patient Register. Positive predictive values have been reported to be about 90% for rheumatoid arthritis and multiple sclerosis, and in the range of 70–90% for subtypes of inflammatory bowel disease.^36^^,^^41^, ^42^, ^43^

#### Outcomes

The primary outcome was all-cause mortality in offspring from birth through young adulthood (up to 23 years of age), ascertained from the Cause of Death Register. Secondary outcomes included deaths classified as natural and unnatural (external) causes. Natural causes of death were further categorized into 12 subgroups, including infectious and parasitic diseases, malignant neoplasms, endocrine, nutritional and metabolic disorders, mental and behavioral disorders, diseases of the nervous system and sense organs, circulatory system diseases, respiratory system diseases, digestive system diseases, musculoskeletal or connective tissue disorders, genitourinary system diseases, and certain conditions originating in the perinatal period, and congenital malformations and chromosomal abnormalities. Corresponding ICD-10 codes used to define each cause-of-death category are provided in Supplementary Table S2.

#### Other variables

Potential confounding factors were selected a priori based on their association to maternal autoimmune diseases and mortality in offspring and included parental sociodemographic background and medical history, and maternal pre-pregnancy health status. Information on maternal age at delivery, parity, marital status, country of birth, and educational level was obtained from nationwide Swedish registers, including the MBR, the Total Population Register, and the Education Register. Maternal anthropometric and lifestyle data recorded in early pregnancy, including height, weight, and smoking habits, were retrieved from the MBR, and body mass index was calculated as kilograms divided by meters squared. Parental medical histories any time before the index childbirth were identified through the Patient Register and included type 2 diabetes, hypertensive disorders, cardiovascular disease, and psychiatric disorders in both mothers and fathers. In addition, family history of diabetes and cardiovascular disease among maternal relatives was assessed using information from the Multi-Generation Register linked with diagnostic data from the Patient Register; these variables were based on available lifetime register information rather than being restricted to the period before childbirth. We also considered birth-related characteristics that may represent intermediate pathways linking maternal autoimmune diseases to offspring mortality. These included preterm birth (<37 completed gestational weeks) and birth weight for gestational age, classified according to Swedish population-based reference curves for fetal growth as small for gestational age (below the 10th percentile) or large for gestational age (above the 90th percentile).^44^ These variables were evaluated in mediation analyses rather than included as confounders in the primary models. Detailed definitions and coding of all covariates are presented in Table 1.

**Table 1:** Baseline characteristics of the exposed and unexposed groups.

Study variables	Exposed to maternal autoimmune diseases	
	No (n = 1,365,599)	Yes (n = 90,046)
Offspring sex		
Male	702,190 (51.42)	46,138 (51.24)
Female	663,409 (48.58)	43,908 (48.76)
Calendar year of birth		
2001–2005	459,201 (33.63)	17,904 (19.88)
2006–2010	502,194 (36.77)	32,584 (36.19)
2011–2014	404,204 (29.60)	39,558 (43.93)
Birth weight for gestational age		
Appropriate	1,079,419 (79.04)	66,598 (73.96)
Small	138,930 (10.17)	9494 (10.54)
Large	144,073 (10.55)	13,743 (15.26)
Missing	3177 (0.23)	211 (0.23)
Preterm birth		
No	1,287,200 (94.26)	81,786 (90.83)
Yes	77,801 (5.70)	8221 (9.13)
Missing	598 (0.04)	39 (0.04)
Autoimmune diseases in offspring		
No	1,308,512 (95.82)	84,283 (93.60)
Yes	57,087 (4.18)	5763 (6.40)
Mother's country of birth		
Sweden	1,064,279 (77.93)	75,196 (83.51)
Other country	299,757 (21.95)	14,731 (16.36)
Missing	1563 (0.11)	119 (0.13)
Maternal age at the time of the index birth (years)		
≤19	21,669 (1.59)	807 (0.90)
20–24	176,241 (12.91)	8282 (9.20)
25–29	408,766 (29.93)	23,301 (25.88)
30–34	710,920 (52.06)	52,549 (58.36)
≥35	48,003 (3.52)	5107 (5.67)
Parity of the index birth		
1	600,997 (44.01)	37,337 (41.46)
2	499,364 (36.57)	33,990 (37.75)
≥3	265,238 (19.42)	18,719 (20.79)
Highest maternal education level at the time of the index birth		
Primary or lower secondary	148,814 (10.90)	8591 (9.54)
Upper secondary	545,094 (39.92)	35,338 (39.24)
Bachelor or higher	640,881 (46.93)	45,269 (50.27)
Missing	30,810 (2.26)	848 (0.94)
Maternal marital status at childbirth		
Yes	486,694 (35.64)	30,199 (33.54)
No	791,678 (57.97)	52,581 (58.39)
Missing	87,227 (6.39)	7266 (8.07)
Maternal smoking in early pregnancy		
No	1,208,352 (88.49)	80,143 (89.0)
Yes	97,308 (7.13)	6047 (6.72)
Missing	59,939 (4.39)	3856 (4.28)
Maternal body-mass index in early pregnancy (kg/m^2^)		
Underweight (<18.5)	30,160 (2.21)	1831 (2.03)
Normal weight (18.5–24.9)	756,825 (55.42)	46,778 (51.95)
Overweight (25.0–29.9)	307,397 (22.51)	21,017 (23.34)
Obesity (≥30.0)	144,257 (10.56)	12,921 (14.35)
Missing	126,960 (9.30)	7499 (8.33)
Maternal hypertensive disorders of pregnancy		
No	1,307,006 (95.71)	83,267 (92.47)
Yes	58,593 (4.29)	6779 (7.53)
Maternal type 2 diabetes before childbirth		
No	1,364,416 (99.91)	87,160 (96.79)
Yes	1183 (0.09)	2886 (3.21)
Maternal cardiovascular diseases before childbirth		
No	1,255,338 (91.93)	77,511 (86.08)
Yes	110,261 (8.07)	12,535 (13.92)
Mother's family history of diabetes		
No	965,778 (70.72)	66,180 (73.50)
Yes	165,473 (12.12)	13,047 (14.49)
Missing	234,348 (17.16)	10,819 (12.01)
Mother's family history of cardiovascular diseases		
No	708,385 (51.87)	44,924 (49.89)
Yes	421,936 (30.90)	34,255 (38.04)
Missing	235,278 (17.23)	10,867 (12.07)
Paternal age at the time of the index birth (years)		
≤19	163,860 (12.0)	12,486 (13.87)
20–24	243,917 (17.86)	18,542 (20.59)
25–29	309,073 (22.63)	21,745 (24.15)
30–34	195,764 (14.34)	11,727 (13.02)
≥35	137,288 (10.05)	7466 (8.29)
Missing	315,697 (23.12)	18,080 (20.08)
Father's country of birth		
Sweden	983,297 (72.0)	68,275 (75.82)
Other country	66,605 (4.88)	3691 (4.10)
Missing	315,697 (23.12)	18,080 (20.08)
Highest paternal education level at the time of the index birth		
Primary and lower secondary	103,075 (7.55)	6512 (7.23)
Upper secondary	676,724 (49.56)	45,329 (50.34)
Bachelor or higher	268,607 (19.67)	20,045 (22.26)
Missing	317,193 (23.23)	18,160 (20.17)
Paternal marital status at childbirth		
Yes	904,348 (66.22)	64,280 (71.39)
No	145,475 (10.65)	7682 (8.53)
Missing	315,776 (23.12)	18,084 (20.08)
Paternal cardiovascular diseases before childbirth		
No	1,034,128 (75.73)	70,936 (78.78)
Yes	15,774 (1.16)	1030 (1.14)
Missing	315,697 (23.12)	18,080 (20.08)
Father's type 2 diabetes before childbirth		
No	1,048,245 (76.76)	71,796 (79.73)
Yes	2488 (0.18)	225 (0.25)
Missing	314,866 (23.06)	18,025 (20.02)

### Statistical analyses

We studied the association between maternal autoimmune diseases during pregnancy and offspring all-cause and cause-specific mortality using Cox proportional hazards regression, with offspring age as the underlying time scale. For cause-specific mortality analyses, deaths from causes other than the cause of interest were treated as competing events, and we estimated cause-specific hazard ratios (HRs). These estimates represent the relative instantaneous risk of death from a specific cause among individuals at risk and do not directly quantify the cumulative incidence of that cause of death. The proportional hazards assumption was assessed using Schoenfeld residuals. To account for potential time-varying associations, we additionally fitted a flexible parametric survival model with a time-varying effect of maternal autoimmune diseases. Separate models were fitted for any maternal autoimmune disease, disease groups, and individual autoimmune diseases when the number of events allowed. In model 1, we controlled for offspring age (as the underlying time scale in the Cox regression models). In model 2, we also controlled for offspring birth year, parity, maternal age, country of birth, education, and marital status at delivery. In model 3, we further controlled for maternal smoking in early pregnancy and pre-existing maternal cardiovascular diseases and type 2 diabetes.

To explore the potential role of shared familial confounding, we conducted (1) negative control analyses using paternal autoimmune diseases during the partner's pregnancy, and (2) cousin analyses comparing maternal cousins (offspring of sisters) using stratified Cox models. The paternal negative control analysis assumes that paternal autoimmune diseases share familial genetic and environmental confounding structures with maternal autoimmune diseases but cannot influence offspring through intrauterine pathways, although this assumption cannot be directly verified. Differences between the maternal-offspring and paternal-offspring associations with mortality for the same autoimmune diseases were quantified and statistically tested using the Wald test. The cousin analyses compared cousins who were discordant for exposure to maternal autoimmune diseases, while stratifying on the maternal grandmother to account for factors shared within the extended family. By comparing sisters' offspring from different pregnancies, this design partially controls for shared genetic background and early-life familial environment (around 25%) while preserving differences in pregnancy-specific maternal exposures.

As maternal autoimmune diseases have been shown to be associated with the risk of adverse birth outcomes such as preterm birth, small for gestational age, large for gestational age, and congenital heart disease,^16^, ^17^, ^18^, ^19^, ^20^, ^21^, ^22^, ^23^ which in turn are associated with increased risks of mortality in offspring,^24^, ^25^, ^26^, ^27^, ^28^ we evaluated whether these factors may mediate the studied association. The mediation analyses were conducted using the counterfactual framework described by Valeri and VanderWeele^45^ and implemented in R using the CMAverse package, estimating total natural direct and indirect effects along with the proportion mediated. The analyses were based on a prespecified causal framework and assumed no substantial unmeasured exposure-mediator, mediator-outcome, or exposure-outcome confounding after adjustment for measured covariates (Supplementary Figure S1). Each adverse birth outcome was evaluated in a separate single-mediator model; therefore, mediated effects should not be interpreted as additive because these mediators were correlated.

Analyses stratified by sex and by small for gestational age were performed to assess effect modification by offspring sex and small for gestational age. In addition, we evaluated whether the associations differed across developmental stages by stratifying follow-up into predefined offspring age periods (0–1, 2–14, 15–18, and >18 years). An interaction between maternal autoimmune diseases and offspring age period was formally tested using a Cox model including an exposure-by-age-period interaction term.

Several sensitivity analyses were performed to evaluate the robustness of the findings. First, to further evaluate the potential contribution of familial susceptibility and additional maternal health factors, we adjusted for maternal family history of diabetes, maternal family history of cardiovascular disease, maternal body mass index in early pregnancy, and paternal autoimmune diseases before childbirth in addition to the covariates in our main models in study participants with available data on these measures. Second, to assess whether the observed associations were explained by specific pregnancy-related or offspring-related conditions, we repeated analyses excluding offspring with maternal hypertensive disorders of pregnancy or offspring with autoimmune diseases, respectively. These analyses were prespecified as sensitivity analyses rather than mediation analyses because hypertensive disorders of pregnancy could not always be temporally ordered after maternal autoimmune disease, whereas associations between maternal and offspring autoimmune diseases may be strongly influenced by shared genetic and familial environmental factors that could not be fully controlled. Third, to reduce potential misclassification of autoimmune disease status, we restricted analyses to mothers with at least two recorded diagnoses for a given autoimmune disease. Fourth, we further restricted analyses to mothers with only one autoimmune disease diagnosis to minimize overlap between autoimmune conditions and enhance exposure specificity. Finally, to account for within-family correlation arising from siblings born to the same mother, we repeated the main analyses using robust sandwich variance estimators clustered on maternal identity.

In additional analyses, we redefined exposure to maternal autoimmune diseases as (1) as being diagnosed within three years postpartum, to identify potential preclinical autoimmune diseases that may have been present during pregnancy but diagnosed later, or (2) being diagnosed more than three years after childbirth, to explore whether associations extended beyond the pregnancy period and could reflect broader maternal or familial factors not related to pregnancy, e.g., growing up with a mother with a chronic medical condition.

Analyses were performed in R version 4.3.1.

### Role of funding source

The funders had no role in the study design, data collection, data analysis, interpretation of the data, or writing the report.

## Results

### Population characteristics

The study included 1,455,645 offspring, of whom 90,046 (6.2%) were born to mothers with autoimmune diseases. The distribution of offspring sex was similar between exposed and unexposed groups. Compared with unexposed offspring, those exposed to maternal autoimmune diseases were more likely to be born in more recent calendar years and to mothers who were older and had higher educational attainment. Adverse perinatal characteristics were more common among exposed offspring, including a higher prevalence of preterm birth (9.1% vs. 5.7%) and large for gestational age (15.3% vs. 10.6%). Exposed offspring also had a higher proportion of autoimmune diseases during follow-up (6.4% vs. 4.2%). Mothers with autoimmune diseases more frequently had comorbid conditions, including pre-existing type 2 diabetes (3.21% vs. 0.09%), cardiovascular diseases (13.9% vs. 8.1%), and hypertensive disorders of pregnancy (7.5% vs. 4.3%).

### Maternal autoimmune diseases and offspring mortality

During a median follow-up of 15.3 years (interquartile range 11.9–19.0), corresponding to 22,339,560 person-years, 426 and 5728 deaths occurred among exposed and unexposed offspring, respectively. Offspring exposed to maternal autoimmune diseases had a higher cumulative incidence of all-cause mortality than unexposed offspring (Supplementary Figure S2). Overall, maternal autoimmune diseases were associated with a modest but consistent increase in the risk of all-cause mortality in offspring (HR = 1.22, 95% confidence interval (CI) = 1.10–1.36). The association remained stable across different models, with similar estimates observed after adjustment for sociodemographic factors, maternal lifestyle, and pre-existing comorbidities (Fig. 1). Heterogeneity was observed across specific maternal autoimmune diseases. Maternal type 1 diabetes (84 exposed deaths) was associated with an approximately twofold higher risk of offspring mortality (HR 1.99, 95% CI 1.46–2.71). Elevated risk estimates were also observed for connective tissue diseases (25 exposed deaths; HR 1.75, 95% CI 1.14–2.69) and autoimmune thyroiditis (148 exposed deaths; HR 1.19, 95% CI 1.00–1.42), although estimates for several rarer autoimmune diseases were based on small numbers of events. In contrast, little to no association was observed for skin, blood or gastrointestinal autoimmune diseases. These estimates represent an average association over the entire follow-up period. The proportional hazards assumption was not clearly violated for maternal autoimmune diseases based on Schoenfeld residuals (*P* = 0.05). When stratified by offspring age, the association was strongest during infancy (HR 1.21, 95% CI 1.06–1.38), with weaker and less precise estimates during childhood, adolescence and adulthood (Table 2). However, there was no evidence that the association differed across age periods (*P* for interaction = 0.73). Consistent with this pattern, the flexible parametric model showed that the hazard ratio was highest during early follow-up, declined toward the null during childhood, and increased modestly at later ages, although CIs were wide (Supplementary Figure S3).

**Fig. 1:**
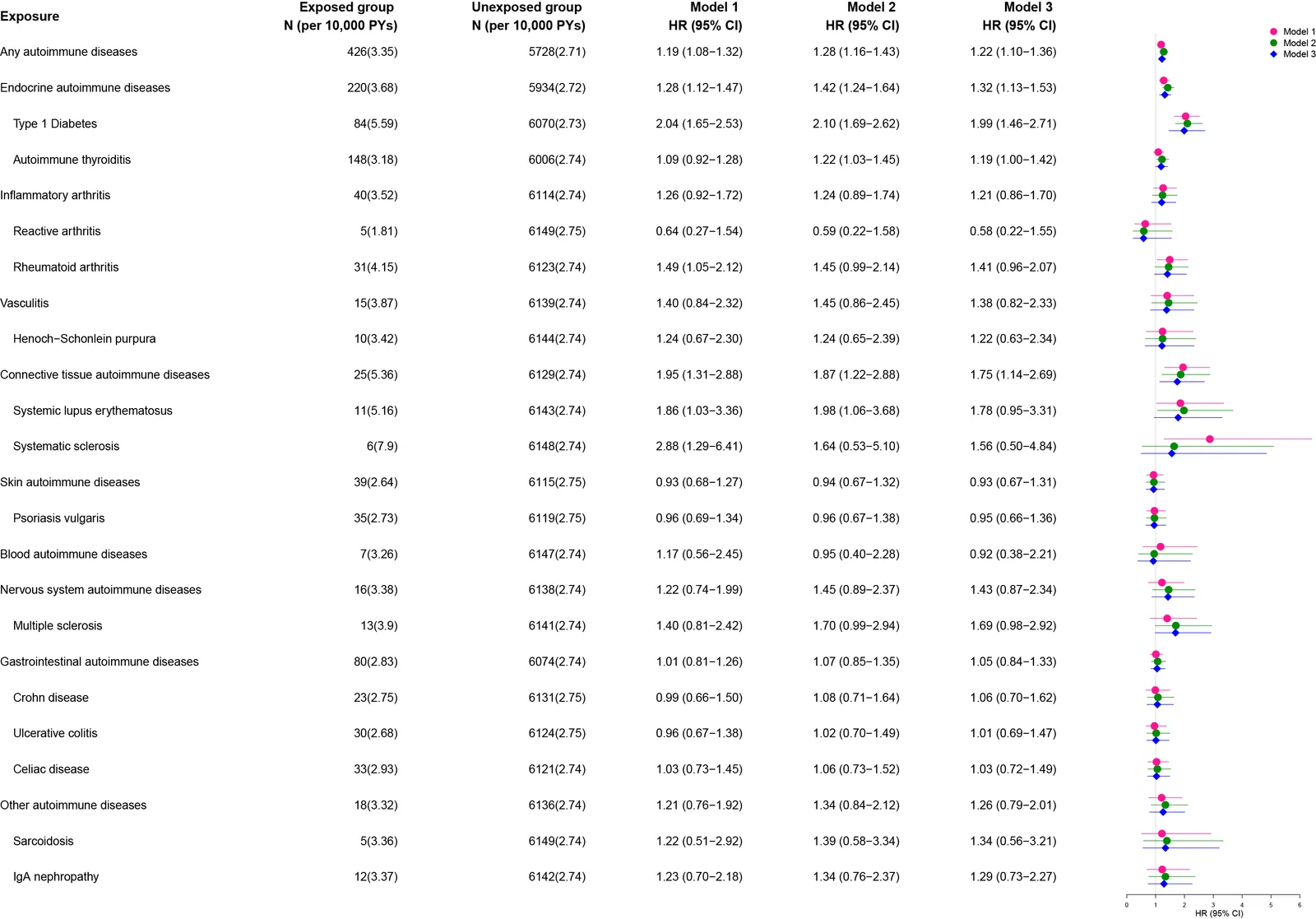
Hazard ratios and 95% confidence intervals for all-cause mortality in offspring according to maternal autoimmune diseases during pregnancy. CI: Confidence interval; HR: Hazard ratio; PY: Person-year. Only autoimmune diseases with ≥5 deaths occurring in the exposed group were included in our analyses. Models 1 were adjusted for offspring age (as the underlying time scale in the Cox regression models); Models 2 were adjusted for offspring's calendar year of birth, parity, maternal age, country of birth, education, and marital status at the time of birth; Models 3 were adjusted for factors in model 2 and for maternal smoking in early pregnancy, maternal cardiovascular diseases and type 2 diabetes before childbirth.

**Table 2:** Hazard ratios and 95% confidence intervals for all-cause mortality in offspring according to maternal autoimmune disease during pregnancy by age period.

Age period (years)	Events (per 10,000 PYs)		HR (95% CI)
	Exposed	Non-exposed	
Infant (≤1)	218 (24.30)	2708 (19.90)	1.21 (1.06–1.38)
Child (2–14)	102 (1.87)	1490 (2.52)	1.11 (0.90–1.38)
Teenage (15–18)	18 (0.48)	351 (0.57)	0.84 (0.48–1.47)
Adult (>18)	14 (0.40)	344 (0.38)	1.17 (0.67–2.06)

CI: Confidence interval; HR: Hazard ratio; PY: Person-year.

Using offspring age as the underlying time scale, analyses were adjusted for offspring's calendar year of birth, parity, maternal age, country of birth, education, and marital status at the time of birth, maternal smoking in early pregnancy, maternal cardiovascular diseases and type 2 diabetes before childbirth.

The excess mortality associated with maternal autoimmune diseases was primarily driven by deaths due to natural causes (HR 1.26, 95% CI 1.12–1.42) (Supplementary Table S3). Among natural causes, the strongest associations were observed for certain conditions originating in the perinatal period (HR 1.43, 95% CI 1.18–1.72) (Supplementary Table S4) and congenital malformations and chromosomal abnormalities (HR 1.29, 95% CI 1.04–1.62) (Supplementary Table S5). In contrast, little to no association was observed for other major causes of death, including infections, malignancies, and diseases of the nervous or circulatory systems (Supplementary Table S6), and no clear association was observed for deaths due to unnatural causes (Supplementary Table S7).

### Family-based analyses

We observed no clear evidence for the association between paternal autoimmune diseases and all-cause (Table 3) or cause-specific mortality (Supplementary Table S8). The association between maternal autoimmune disease and all-cause mortality was stronger than that corresponding to paternal autoimmune disease (426 maternal vs. 164 paternal deaths), with a ratio of HR of 1.26 (95% CI 1.03–1.53) in case of any autoimmune diseases. For specific autoimmune disease, the association between maternal type 1 diabetes and all-cause mortality was stronger than that corresponding to paternal type 1 diabetes (84 maternal vs 29 paternal deaths), with a ratio of HR of 2.73 (95% CI 1.30–5.73) (Table 3). Similar patterns were found for mortality due to specific causes, although estimates for individual autoimmune diseases were generally less precise because of limited event numbers (Supplementary Table S8).

**Table 3:** Hazard ratios and 95% confidence intervals for all-cause mortality in offspring according to paternal autoimmune diseases during the partner's pregnancy.

Exposure	Paternal events (/10,000 PYs)		Paternal HR (95% CI)^a^	Maternal events (/10,000 PYs)		Maternal HR (95% CI)^b^	Maternal-paternal comparison^c^	
	Exposed	Non-exposed		Exposed	Non-exposed		Ratio (95% CI)	*P* value
Any autoimmune diseases	164 (2.39)	4103 (2.44)	0.97 (0.82–1.14)	335 (3.27)	3932 (2.39)	1.36 (1.20–1.53)	1.40 (1.14–1.72)	0.001
Endocrine autoimmune diseases	30 (2.29)	4237 (2.44)	0.76 (0.40–1.45)	169 (3.74)	4098 (2.40)	1.55 (1.30–1.85)	2.04 (1.05–3.98)	0.036
Type 1 diabetes	29 (2.27)	4238 (2.44)	0.73 (0.37–1.43)	64 (5.39)	4203 (2.42)	2.61 (1.75–3.90)	3.58 (1.63–7.84)	0.001
Autoimmune thyroiditis	29 (2.27)	4238 (2.44)	0.73 (0.37–1.43)	112 (3.23)	4155 (2.42)	1.36 (1.11–1.66)	1.86 (0.92–3.77)	0.084
Inflammatory arthritis	18 (2.19)	4249 (2.44)	0.90 (0.57–1.43)	33 (3.43)	4234 (2.43)	1.35 (0.93–1.94)	1.50 (0.83–2.70)	0.177
Reactive arthritis	10 (2.84)	4257 (2.44)	1.16 (0.62–2.16)	5 (2.16)	4262 (2.44)	0.77 (0.29–2.06)	0.66 (0.21–2.12)	0.489
Rheumatoid arthritis	9 (3.27)	4258 (2.44)	1.36 (0.71–2.62)	25 (3.93)	4242 (2.43)	1.54 (1.01–2.34)	1.13 (0.52–2.46)	0.754
Vasculitis autoimmune diseases	12 (3.25)	4255 (2.44)	1.36 (0.77–2.39)	13 (3.88)	4254 (2.44)	1.50 (0.85–2.65)	1.10 (0.49–2.46)	0.811
Henoch-Schonlein purpura	9 (3.15)	4258 (2.44)	1.34 (0.70–2.57)	9 (3.54)	4258 (2.44)	1.36 (0.68–2.73)	1.01 (0.39–2.63)	0.976
Connective tissue autoimmune diseases	6 (2.86)	4261 (2.44)	1.15 (0.52–2.57)	17 (4.41)	4250 (2.43)	1.76 (1.08–2.88)	1.53 (0.60–3.91)	0.374
Skin autoimmune diseases	30 (2.30)	4237 (2.44)	0.96 (0.67–1.37)	31 (2.48)	4236 (2.44)	0.98 (0.67–1.43)	1.02 (0.61–1.72)	0.938
Psoriasis vulgaris	28 (2.40)	4239 (2.44)	1.00 (0.69–1.45)	29 (2.66)	4238 (2.44)	1.03 (0.70–1.53)	1.03 (0.60–1.77)	0.914
Gastrointestinal autoimmune diseases	47 (2.33)	4220 (2.44)	0.93 (0.69–1.25)	67 (2.72)	4200 (2.43)	1.11 (0.86–1.43)	1.19 (0.81–1.76)	0.375
Crohn disease	18 (2.65)	4249 (2.44)	1.04 (0.64–1.67)	16 (2.23)	4251 (2.44)	0.92 (0.56–1.53)	0.88 (0.44–1.77)	0.729
Ulcerative colitis	25 (2.31)	4242 (2.44)	0.96 (0.65–1.43)	26 (2.69)	4241 (2.44)	1.08 (0.72–1.63)	1.13 (0.64–1.98)	0.684
Celiac disease	10 (2.10)	4257 (2.44)	0.80 (0.42–1.54)	29 (2.90)	4238 (2.44)	1.15 (0.78–1.69)	1.44 (0.67–3.06)	0.347
Other autoimmune diseases	23 (2.82)	4244 (2.44)	1.13 (0.75–1.70)	12 (2.74)	4255 (2.44)	1.14 (0.65–2.02)	1.01 (0.50–2.03)	0.980
IgA nephropathy	20 (3.61)	4247 (2.43)	1.46 (0.94–2.26)	7 (2.40)	4260 (2.44)	1.00 (0.48–2.10)	0.68 (0.29–1.62)	0.388

CI: Confidence interval; HR: Hazard ratio; PY: Person-year.

Only autoimmune diseases with ≥5 deaths occurring in the exposed group were included in our analyses.

a Using offspring age as the underlying time scale, analyses were adjusted for offspring's calendar year of birth, paternal age, country of birth, education, and marital status at the time of birth, paternal cardiovascular diseases and type 2 diabetes before childbirth.

b Maternal associations were estimated using the same analytic sample (n = 1122754); analyses were adjusted for offspring's calendar year of birth, maternal country of birth, parity, age, education, and marital status at the time of birth, maternal smoking in early pregnancy, maternal cardiovascular diseases and type 2 diabetes before childbirth.

c The maternal-to-paternal association ratio was defined as the adjusted hazard ratio for maternal autoimmune disease divided by the corresponding adjusted hazard ratio for paternal autoimmune disease. The 95% confidence intervals were calculated using the Wald method.

We further performed cousin analyses to account for unmeasured familial confounding. The associations between maternal autoimmune diseases and all-cause mortality in offspring were generally attenuated (HR 1.04, 95% CI 0.81–1.33). Estimates for specific autoimmune diseases were generally less precise because of the substantially smaller number of informative cousin comparisons; for example, the HR for maternal type 1 diabetes was 2.63 (95% CI 1.19–5.79). Analyses for cause-specific mortality showed similar patterns (Table 4; Supplementary Table S9).

**Table 4:** Hazard ratios and 95% confidence intervals for all-cause mortality in offspring according to maternal autoimmune diseases during pregnancy in cousin analyses.

Exposure	Events (per 10,000 PYs)		Informative cousin clusters^a^	HR (95% CI)
	Exposed	Non-exposed		
Any autoimmune diseases	239 (3.55)	2584 (2.39)	388	1.04 (0.81–1.33)
Endocrine autoimmune diseases	120 (4.12)	2703 (2.41)	198	1.19 (0.84–1.68)
Type 1 diabetes	44 (5.80)	2779 (2.43)	53	2.63 (1.19–5.79)
Autoimmune thyroiditis	82 (3.65)	2741 (2.43)	157	1.01 (0.69–1.48)
Inflammatory arthritis	25 (3.98)	2798 (2.45)	39	1.08 (0.49–2.40)
Rheumatoid arthritis	19 (4.60)	2804 (2.45)	28	1.26 (0.50–3.16)
Vasculitis autoimmune diseases	8 (3.62)	2815 (2.45)	12	0.17 (0.02–1.57)
Henoch-Schonlein purpura	6 (3.49)	2817 (2.45)	10	0.22 (0.02–2.36)
Connective tissue autoimmune diseases	13 (5.10)	2810 (2.45)	24	0.87 (0.35–2.16)
Systemic lupus erythematosus	8 (6.92)	2815 (2.45)	10	0.92 (0.24–3.48)
Skin autoimmune diseases	20 (2.42)	2803 (2.45)	57	0.57 (0.27–1.20)
Psoriasis vulgaris	20 (2.78)	2803 (2.45)	54	0.63 (0.29–1.33)
Nervous autoimmune diseases	14 (5.43)	2809 (2.45)	19	1.63 (0.57–4.65)
Multiple sclerosis	11 (6.14)	2812 (2.45)	14	1.47 (0.41–5.27)
Gastrointestinal autoimmune diseases	47 (2.83)	2776 (2.45)	78	0.97 (0.58–1.63)
Crohn disease	12 (2.47)	2811 (2.45)	22	0.48 (0.16–1.44)
Ulcerative colitis	20 (3.06)	2803 (2.45)	29	1.20 (0.50–2.88)
Celiac disease	18 (2.65)	2805 (2.45)	35	1.15 (0.54–2.44)
Other autoimmune diseases	7 (2.42)	2816 (2.45)	18	1.09 (0.33–3.58)

CI: Confidence interval; HR: Hazard ratio; PY: Person-year.

Only autoimmune diseases with ≥5 deaths occurring in the exposed group were included in our analyses.

Using offspring age as the underlying time scale, analyses were adjusted for offspring's calendar year of birth, parity, maternal age, country of birth, education, and marital status at the time of birth, maternal smoking in early pregnancy, maternal cardiovascular diseases and type 2 diabetes before childbirth.

a Informative cousin clusters were defined as cousin clusters that were discordant for both the maternal autoimmune disease exposure (ie, including at least 1 exposed and 1 unexposed offspring) and offspring all-cause mortality (i.e., including at least 1 offspring who died and 1 who did not).

### Mediation analyses

We next investigated whether the associations between maternal autoimmune diseases and offspring mortality were mediated by adverse birth outcomes (Table 5). Preterm birth showed evidence of mediation, with a mediated effect greater than 1 across outcomes. Although the estimated proportion mediated was relatively large, CIs were wide for several birth outcomes, indicating substantial uncertainty in the magnitude of mediation (Table 5). Small for gestational age accounted for a smaller but notable proportion of the effect, with 16.8% mediation for all-cause mortality and 24.4% for perinatal conditions. Congenital heart disease mediated a smaller proportion of the effect, with 7.5% mediation for all-cause mortality and 22.5% for congenital anomalies. In contrast, large for gestational age did not meaningfully mediate the associations.

**Table 5:** Hazard ratios and 95% confidence intervals for offspring mortality according to any maternal autoimmune disease during pregnancy mediated by preterm birth, small or large for gestational age and congenital heart disease.

Mediator	Number of participants	Cause of death	HR (95% CI)			Proportion mediated (%)	*P* value for the interaction^a^
			Total effect	TNDE	TNIE		
Preterm birth	1,129,756	All cause	1.24 (1.10–1.39)	1.09 (0.97–1.23)	1.12 (1.09–1.14)	53.46 (35.17–124.33)	0.41
		Natural cause	1.26 (1.07–1.43)	1.08 (0.91–1.21)	1.14 (1.11–1.17)	57.03 (38.16–184.54)	0.15
		Congenital anomalies	1.34 (1.05–1.69)	1.15 (0.91–1.47)	1.12 (1.08–1.17)	42.79 (21.62–147.53)	0.59
		Conditions originating in the perinatal period	1.37 (1.13–1.64)	1.05 (0.86–1.25)	1.25 (1.20–1.31)	75.05 (50.03–176.95)	0.59
Small for gestational age	999,880	All cause	1.23 (1.10–1.41)	1.20 (1.07–1.36)	1.03 (1.02–1.04)	16.82 (10.38–35.70)	0.02
		Natural cause	1.24 (1.07–1.43)	1.20 (1.03–1.38)	1.04 (1.03–1.05)	17.62 (9.84–55.02)	0.27
		Congenital anomalies	1.35 (1.06–1.74)	1.28 (1.00–1.64)	1.05 (1.03–1.06)	16.79 (8.57–87.13)	0.94
		Conditions originating in the perinatal period	1.24 (1.00–1.54)	1.20 (0.96–1.48)	1.05 (1.03–1.07)	24.40 (7.65–272.47)	0.09
Large for gestational age	1,020,774	All cause	1.17 (1.03–1.37)	1.17 (1.03–1.37)	1.00 (0.99–1.01)	−0.46 (−8.51 to 5.94)	0.96
		Natural cause	1.22 (1.02–1.41)	1.22 (1.02–1.41)	1.00 (0.99–1.01)	0.49 (−6.22 to 9.35)	0.62
		Congenital anomalies	1.42 (0.99–1.87)	1.42 (0.99–1.87)	1.00 (0.98–1.01)	−1.44 (−10.24 to 6.81)	0.53
		Conditions originating in the perinatal period	1.16 (0.86–1.55)	1.16 (0.86–1.56)	1.01 (1.00–1.03)	10.09 (−43.80 to 74.90)	0.28
Congenital heart disease	1,130,256	All cause	1.25 (1.09–1.38)	1.22 (1.07–1.36)	1.01 (1.01–1.02)	7.48 (4.37–18.94)	0.74
		Natural cause	1.26 (1.12–1.43)	1.23 (1.09–1.39)	1.02 (1.01–1.03)	8.81 (4.74–17.86)	0.58
		Congenital anomalies	1.33 (1.06–1.65)	1.25 (1.00–1.54)	1.06 (1.04–1.09)	22.54 (11.46–84.53)	0.69
		Conditions originating in the perinatal period	1.36 (1.10–1.64)	1.35 (1.10–1.64)	1.00 (1.00–1.01)	0.23 (−2.07 to 3.32)	0.67

CI: Confidence interval; HR: Hazard ratio; TNDE: Total natural direct effect; TNIE: Total natural indirect effect (Mediated effect).

Using offspring age as the underlying time scale, analyses were adjusted for offspring's calendar year of birth, maternal country of birth, parity, age, education, and marital status at the time of birth, maternal smoking in early pregnancy, maternal cardiovascular diseases and type 2 diabetes before childbirth.

The numbers of participants vary among analyses corresponding to different mediators because of the differences in the number of individuals with missing data on each mediator and exposure-mediator interactions.

a *P* value for the exposure-mediator interactions. Wald tests were used to calculate two-sided *P* values.

### Secondary analyses

In sex-stratified analyses, the associations between maternal autoimmune diseases and offspring all-cause mortality tended to be somewhat more pronounced in males. For instance, any maternal autoimmune disease was associated with a 28% increased risk in male offspring (HR 1.28, 95% CI 1.11–1.48) vs a 14% increase in female offspring (HR 1.14, 95% CI 0.97–1.35). The point estimates for endocrine autoimmune diseases, type 1 diabetes, autoimmune thyroiditis, and connective tissue diseases were particularly higher in males compared to females (Supplementary Table S10). The associations between maternal autoimmune diseases and offspring all-cause mortality were broadly similar across small for gestational age strata (Supplementary Table S11).

Additional adjustment for maternal family history of diabetes (Supplementary Table S12), paternal autoimmune diseases (Supplementary Table S13), maternal early-pregnancy body mass index (Supplementary Table S14), or maternal family history of cardiovascular diseases (Supplementary Table S15) did not materially change the associations between maternal autoimmune diseases and offspring mortality in the subsets of study participants with available data on these variables. When we restricted to offspring of mothers without hypertensive disorders of pregnancy (Supplementary Table S16) and offspring without autoimmune diseases (Supplementary Table S17), the associations between maternal autoimmune diseases and offspring all-cause and natural-cause mortality remained largely consistent with those in the main analysis. Defining maternal autoimmune diseases based on at least two diagnosis records for each condition did not materially change the associations with offspring mortality (Supplementary Table S18). Restricting to mothers with only a single autoimmune disease yielded similar HRs as the main analysis (Supplementary Table S19). The results were virtually unchanged after accounting for within-family correlation by clustering on maternal identity (Supplementary Table S20). Conducting analyses with the exposure defined as maternal autoimmune diseases diagnosed within three years after childbirth resulted in associations with offspring mortality similar to those in the main analysis (Supplementary Table S21). Maternal autoimmune diseases diagnosed after the child reached three years of age showed results consistent with the primary findings (Supplementary Table S22).

## Discussion

In this large, nationwide cohort study, maternal autoimmune diseases were associated with a modest but consistent increase in offspring mortality, with the excess risk largely confined to the first year of life. The association was largely driven by deaths related to conditions originating in the perinatal period and congenital malformations. Among specific autoimmune diseases, maternal type 1 diabetes was associated with an approximately twofold higher risk of offspring mortality. Cousin analyses yielded generally attenuated but less precise estimates, consistent with a possible contribution of shared genetic or familial environmental factors. Paternal autoimmune diseases showed little association with offspring mortality. These together supported the specificity of maternal associations and are consistent with the hypothesis that shared familial factors alone may not fully explain the observed associations and support a contribution of intrauterine mechanisms, while acknowledging that extrauterine mechanisms cannot be excluded. Mediation analyses suggested that adverse perinatal outcomes, including preterm birth and small for gestational age, may contribute to the observed association.

Existing evidence on maternal autoimmune diseases and offspring survival has been largely confined to specific conditions and early-life outcomes, with heterogeneous findings.^30^, ^31^, ^32^, ^33^, ^34^, ^35^ Studies of maternal type 1 diabetes have reported increased long-term mortality in offspring, alongside markedly elevated risks of adverse perinatal outcomes, suggesting that metabolic and intrauterine disturbances may have lasting consequences.^30^, ^31^, ^32^ Similarly, offspring of mothers with systemic lupus erythematosus have been shown to experience increased early-life mortality and morbidity.^33^ By contrast, investigations of celiac disease have generally found little or no association with offspring mortality, despite evidence of increased susceptibility to infections and other morbidities.^34^^,^^35^ Our findings extend this literature in several important ways. First, in a large, population-based cohort with follow-up into early adulthood, we found that maternal autoimmune diseases as a broad group are associated with a modest but consistent increase in offspring mortality risk, concentrated in early life and largely driven by deaths due to conditions originating in the perinatal period and congenital malformations. This pattern suggests that maternal autoimmune diseases primarily influence survival through disruption of fetal and early postnatal development, rather than through persistent increases in mortality risk across later childhood and early adulthood. Second, we identified substantial heterogeneity across disease categories, with particularly elevated risks observed for endocrine autoimmune diseases, particularly type 1 diabetes. However, estimates for several rarer autoimmune diseases were based on few events and should therefore be interpreted cautiously. Third, family-based and paternal negative control analyses were consistent with a contribution of intrauterine mechanisms, suggesting that shared familial factors alone may not fully explain the observed associations. However, paternal comparisons cannot exclude maternal-specific extrauterine mechanisms, including postnatal pathways that differ between mothers and fathers. Finally, by integrating cause-specific mortality, family-based approaches, and mediation analyses, we provided mechanistic insight into how maternal autoimmune diseases may influence early-life survival, linking maternal immune dysregulation to adverse perinatal outcomes and increased offspring vulnerability. However, these analyses should be interpreted as complementary rather than definitive evidence regarding intrauterine mechanisms.

Several complementary mechanisms likely contribute to the observed associations, encompassing intrauterine biological effects, perinatal pathways, and familial influences. A central pathway involves an altered intrauterine environment in pregnancies complicated by maternal autoimmune diseases. Chronic maternal inflammation, immune dysregulation, and transplacental transfer of autoantibodies may impair placental function and fetal development, leading to structural and physiological alterations that increase early-life vulnerability.^11^^,^^46^ Notably, our cohort included births from 2001 onwards, reflecting a period in which the clinical management of autoimmune diseases during pregnancy has substantially improved. The persistence of elevated early-life mortality in this contemporary birth cohort suggests that the observed associations are unlikely to be fully explained by suboptimal clinical care alone. However, differences in disease severity, treatment, and other maternal factors may also have contributed and their influence cannot be excluded in this observational study.

Consistent with this pathway, our mediation analyses showed that adverse perinatal outcomes—particularly preterm birth and small for gestational age—account for a substantial portion of the excess risk, with the observed mortality increase being largely driven by deaths related to conditions originating in the perinatal period and congenital malformations. The stronger associations observed for endocrine autoimmune diseases, especially type 1 diabetes, likely reflect combined effects of metabolic and immune disturbances on placental function and fetal growth.^47^ In contrast, weaker associations for other autoimmune conditions suggested disease-specific mechanisms. Importantly, maternal autoimmune diseases were not associated with unnatural deaths in offspring, providing a potential outcome-based negative control that reinforces the specificity of the observed associations to biologically mediated early-life mortality. This pattern is consistent with an important contribution of mechanisms primarily involving intrauterine effects and adverse perinatal development, although extrauterine mechanisms related to pregnancy, delivery, or the postnatal period may also contribute to the observed associations.

Finally, paternal and cousin comparison analyses indicate that shared genetic and environmental factors alone are unlikely to fully explain the observed associations. However, the reduced precision of the cousin analyses warrants cautious interpretation. Associations remained similar when maternal autoimmune diseases were defined by diagnoses within three years postpartum or more than three years after childbirth, suggesting that the observed associations may not be restricted to diseases clinically recognized before or during pregnancy and may also reflect broader maternal or familial factors. Taken together, these findings are consistent with a model in which intrauterine biological processes and adverse perinatal outcomes contribute importantly to the observed associations, with additional contributions from broader familial and other maternal factors. This may highlight the critical window of fetal development as a period when maternal immune health can have lasting consequences on child survival.

These findings may have implications for population health and future clinical research. Maternal autoimmune diseases, particularly endocrine conditions such as type 1 diabetes, were associated with an increased risk of adverse perinatal outcomes and early-life mortality in offspring. The persistence of these associations in a contemporary birth cohort suggests that improved obstetric care alone may not fully eliminate excess risk and highlights the potential importance of maternal immune and metabolic health during pregnancy. Although the observed effect sizes were modest for most autoimmune conditions, the findings may help identify pregnancies and offspring at increased vulnerability and support continued attention to maternal health before and during pregnancy. Further mechanistic and interventional studies are needed before these findings can inform specific clinical recommendations or targeted prevention strategies.

This study has several strengths. The use of a large, nationwide birth cohort with prospectively collected register data enabled near-complete follow-up from birth into early adulthood and minimized selection and recall bias. The broad assessment of maternal autoimmune diseases across multiple organ systems allowed us to move beyond single-disease analyses and to examine heterogeneity in risk. In addition, the incorporation of family-based designs provided a quasi-experimental framework to assess the potential contribution of shared familial factors, while mediation analyses offered insight into intermediate pathways linking maternal disease to offspring mortality.

Several limitations should also be mentioned. First, although we accounted for a wide range of sociodemographic and parental health characteristics, residual confounding from unmeasured factors—such as health behaviors and potential extrauterine mechanisms (e.g., factors related to pregnancy management, delivery, or the postnatal period)—cannot be excluded. In addition, because the cohort was restricted to live births, and then pregnancies ending in early pregnancy loss, termination or stillbirth were not captured. If maternal autoimmune diseases increase the risk of fetal loss, selection of surviving pregnancies may have led to underestimation of the overall impact of maternal autoimmune diseases on offspring health. Furthermore, we were unable to fully account for medication exposures during pregnancy, which may have contributed to some observed associations, particularly congenital malformations, or for the overall burden of maternal multimorbidity. Second, exposure misclassification is possible, particularly because diagnoses made exclusively in primary care, private practice, or milder autoimmune conditions not referred to specialist care may not have been captured. Ascertainment of maternal autoimmune diseases may also have varied by calendar period, with more recent pregnancies potentially having a more complete capture of prior diagnoses. This may have resulted in underascertainment of milder autoimmune diseases and an exposure group containing more severe cases, potentially leading either to attenuation or overestimation of the observed associations depending on the underlying disease severity and healthcare utilization. In addition, although we included a broad range of autoimmune diseases, our exposure definition did not capture the full spectrum of autoimmune conditions. Therefore, our findings should be interpreted within the scope of the diseases included in this study. Third, the limited number of exposed deaths for some specific autoimmune diseases resulted in reduced precision and wide CIs, even in a violation of the asymptotic assumption in some subgroup and cause-specific analyses. Given the multiple disease-specific, cause-specific, and subgroup comparisons performed, these findings should be considered exploratory and hypothesis-generating until replicated in other populations. In addition, paternal negative-control analyses for individual autoimmune diseases were also based on relatively few exposed deaths, and missing (about 23%) information on some paternal characteristics may have further reduced precision. Fourth, although our design provides indirect evidence regarding intrauterine mechanisms, causal inference remains constrained by the observational nature of the study. In particular, the mediation analyses rely on assumptions of no unmeasured exposure-mediator, exposure-outcome, or mediator-outcome confounding, which cannot be fully verified. Unmeasured factors such as maternal disease severity, glycemic control, treatment, and pregnancy complications may have influenced both the mediators and offspring mortality, potentially affecting the mediation estimates. Nonetheless, the consistency of findings across multiple analytic approaches—including alternative exposure definitions, restricted, and sensitivity analyses—supports the robustness of the overall conclusions.

In this nationwide cohort study, maternal autoimmune diseases, particularly endocrine conditions such as type 1 diabetes, were associated with a modestly increased risk of offspring mortality during the first year of life. The excess mortality was primarily attributable to deaths due to conditions originating in the perinatal period and congenital malformations. These findings were consistent with a contribution of intrauterine mechanisms and adverse perinatal pathways, although additional maternal and familial factors may also contribute to the observed associations. These results may be informative for targeted interventions aimed at optimizing maternal health and perinatal care to improve survival outcomes in children at highest risk.

## Contributors

KL acquired funding for the data and supervised the study. XM carried out the data analysis and interpretation. HZ drafted the original manuscript. IJ, EA, JM, YL, XM, and KL contributed to study conception, design, data interpretation, and editing the manuscript. All authors reviewed and approved the final manuscript. XM and KL accessed and verified data and had final responsibility for the decision to submit the manuscript for publication.

## Data sharing statement

Qualified Swedish researchers may request similar data from the register holders after approval from the regional ethical review board and the relevant registry authorities, i.e., the Swedish National Board of Health and Welfare and Statistics Sweden.

## Declaration of interests

EA reports grants or contracts from the Strategic Research Area in Epidemiology at Karolinska Institutet, the Swedish Heart-Lung Foundation, and the Swedish Research Council. EA has received payment for speaking at a symposium sponsored by Boehringer Ingelheim. All other authors declare no competing interests.
