# Supplementary material for: Maternal autoimmune diseases during pregnancy and mortality up to young adulthood in offspring: a register-based cohort study
Source: eClinicalMedicine. 2026 Sep 22;100:104237. doi: 10.1016/j.eclinm.2026.104237 (PMC13651480; doi:10.1016/j.eclinm.2026.104237)
Supplement: Supplementary file 1 [file mmc1.docx]

**Supplementary data**

Supplementary Table S1. The International Classification of Diseases codes used to identify the diagnoses from the Patient Register or the Medical Birth Register

| Disease | | ICD version | | | | | |  |
| --- | --- | --- | --- | --- | --- | --- | --- | --- |
|  |  | ICD-8 | | ICD-9 | | ICD-10 | |  |
| **Autoimmune diseases**† | |  | |  | |  | |  |
|  | **Endocrine autoimmune diseases** | |  | |  | |  | |
|  | Type 1 diabetes | | - | | - | | E10 | |
|  | Autoimmune thyroiditis | | 242.00,242.09,244,245.03 | | 242A,242X,244W,244X,245C,245W | | E03.5, E03.9, E05.0, E05.5, E05.9, E06.3, E06.5 | |
|  | Primary adrenocortical insufficiency | | 255.1 | | 255E | | E27.1, E27.2 | |
|  | Autoimmune polyglandular syndrome | | 258.1 | | 258B | | E31.0 | |
|  | **Inflammatory arthritis** | |  | |  | |  | |
|  | Reactive arthritis (Reiter’s syndrome) | | 099.3, 711.1 | | 099D, 711A | | M02.3, M02.8, M02.9 | |
|  | Rheumatoid arthritis | | 712.00, 712.10, 712.20, 712.39, 712.38 | | 714A, 714B, 714C, 714D, 719D | | M05, M06, M08.0, M08.1, M08.2, M08.3, M08.4 | |
|  | Ankylosing spondylitis | | 712.4 | | 720A | | M45 | |
|  | **Vasculitis** | |  | |  | |  | |
|  | Henoch-Schonlein purpura | | 287 | | 287A | | D69.0 | |
|  | Polyarteritis nodosa and related condition | | 446 | | 446A, 446B | | M30 | |
|  | Thrombotic microangiopathy | | - | | 446G | | M31.1 | |
|  | Granulomatosis with polyangiitis/Wegener granulomatosis | | 446.2 | | 446E | | M31.3 | |
|  | Microscopic polyangiitis | | - | | - | | M31.7 | |
|  | Polymyalgia rheumatica | | 446.30, 446.31, 446.39,717.9 | | 446F, 725 | | M31.5, M31.6, M35.3 | |
|  | **Connective tissue autoimmune diseases** | |  | |  | |  | |
|  | Systemic lupus erythematosus | | 734.1 | | 710A | | M32 | |
|  | Polymyositis/Dermatopolymyositis | | 716.00,716.10 | | 710D,710E | | M33.0, M33.1, M33.2, M33.9 | |
|  | Systematic sclerosis/Scleroderma | | 710.0,734.0 | | 710B | | M34 | |
|  | Sjögren’s disease | | 734.9 | | 710C | | M35.0 | |
|  | Mixed connective tissue disease | | 734.98,734.91,734.99 | | 710X,710W | | M35.1 | |
|  | Behcet′s syndrome | | 136 | | 136B | | M35.2 | |
|  | **Skin autoimmune diseases** | |  | |  | |  | |
|  | Pemphigus vulgaris | | 694 | | 694E | | L10.0 | |
|  | Bullous pemphigoid | | 694.05 | | 694F | | L12 | |
|  | Dermatitis herpetiformis | | 693.99 | | 694A | | L13.0 | |
|  | Psoriasis vulgaris | | 696 | | 696A, 696B | | L40 | |
|  | Alopecia areata | | 704 | | 704A | | L63 | |
|  | Vitiligo | | 709.05 | | 709A | | L80 | |
|  | **Blood autoimmune diseases** | |  | |  | |  | |
|  | Pernicious anemia | | 281 | | 281A | | D51.0 | |
|  | Autoimmune hemolytic anemia | | 283.90, 283.92 | | 283A | | D59.0, D59.1 | |
|  | Idiopathic thrombocytopenic purpura | | 287.3 | | 287D | | D69.3 | |
|  | **Nervous system autoimmune diseases** | |  | |  | |  | |
|  | Acute disseminated encephalomyelitis | | - | | - | | G04 | |
|  | Anti-NMDA receptor encephalitis | | - | | - | | G13.1 | |
|  | Multiple sclerosis | | 340 | | 340 | | G35 | |
|  | Neuromyelitis optica | | 341 | | 341A | | G36 | |
|  | Guillain-Barré syndrome | | 354.01 | | 357A | | G61.0, G61.1, G61.8, G61.9 | |
|  | Myasthenia gravis | | 733 | | 358A | | G70.0 | |
|  | **Gastrointestinal autoimmune diseases** | |  | |  | |  | |
|  | Primary biliary cirrhosis | | - | | 571G | | K74.3 | |
|  | Crohn disease | | 563 | | 555 | | K50 | |
|  | Ulcerative colitis | | 563.10,569.02 | | 556 | | K51 | |
|  | Celiac disease | | 269.00,269.98 | | 579A | | K90.0 | |
|  | **Other autoimmune diseases** | |  | |  | |  | |
|  | Acute rheumatic fever and chorea | | 390, 391, 392 | | 390, 391, 392 | | I00, I01.0, I01.1, I01.2, I01.8, I01.9, I02.0, I02.9 | |
|  | Sarcoidosis | | 135 | | 135 | | D86 | |
|  | Autoimmune hepatitis | | 571.93 | | 571.9 | | K75.4 | |
|  | IgA nephropathy | | 580, 582 | | 580, 582 | | N00, N01, N03, N05 | |
| Cardiovascular diseases | | 390-458 | | 390-549 | | I00-I99 | |  |
| Maternal hypertensive disorders of pregnancy | | 400-404, 63701, 63703, 63704, 63709, 63799, 63710 | | 401-405, 642A, 642B, 642C, 642D, 642E, 642F, 642G, 642H, 642X | | I10-I15, O10, O11, O13, O14, O15, O16 | |  |
| Diabetes mellitus | | 250 | | 250, 648A, 648W | | E10-E14, O24 | |  |
| Psychiatric disorders | | 290-315 | | 290-319 | | F00-F99 | |  |

ICD: International Classification of Diseases. The International Classification of Diseases’ 8^th^ revision was used during 1973-1986, the 9^th^ revision during 1987-1996 and the 10^th^ revision since 1997.

†: The nine groups of autoimmune diseases were defined following the classification used in our previous paper (Eur Heart J. 2026:ehag316. doi: 10.1093/eurheartj/ehag316).

Supplementary Table S2 International Classification of Diseases codes used to classify causes of death

| Cause of Death | ICD-10 |
| --- | --- |
| **All causes of death** | A00-Y89 |
| **Natural death** | A00-Q99 |
| Infectious and parasitic diseases | A00-B99 |
| Malignant neoplasms | C00-C97 |
| Endocrine, nutritional and metabolic diseases | E00-E90 |
| Mental and behavioural disorders | F00-F99 |
| Diseases of the nervous system and the sense organs | G00-H95 |
| Diseases of the circulatory system | I00-I99 |
| Diseases of the respiratory system | J00-J99 |
| Diseases of the digestive system | K00-K93 |
| Diseases of the musculoskeletal system or connective tissue | M00-M99 |
| Diseases of the genitourinary system | N00-N99 |
| Certain conditions originating in the perinatal period | P00-P96 |
| Congenital malformations and chromosomal abnormalities | Q00-Q99 |
| **External (unnatural) causes of morbidity and mortality** | V01-Y98 |

Supplementary Table S3 Hazard ratios and 95% confidence intervals for mortality due to natural causes in offspring according to maternal autoimmune diseases during pregnancy

| Exposure | Events (per 10,000 PYs) | |  | HR (95% CI) | | |
| --- | --- | --- | --- | --- | --- | --- |
|  | Exposed | Non-exposed |  | Model 1 | Model 2 | Model 3 |
| **Any autoimmune diseases** | 336 (2.64) | 4331 (2.05) |  | 1.21 (1.08-1.35) | 1.32 (1.18-1.49) | 1.26 (1.12-1.42) |
| **Endocrine autoimmune diseases** | 176 (2.94) | 4491 (2.06) |  | 1.31 (1.12-1.52) | 1.46 (1.25-1.71) | 1.36 (1.15-1.61) |
| Type 1 diabetes | 68 (4.52) | 4599 (2.06) |  | 2.17 (1.71-2.75) | 2.29 (1.79-2.92) | 2.35 (1.65-3.34) |
| Autoimmune thyroiditis | 118 (2.53) | 4549 (2.07) |  | 1.09 (0.91-1.31) | 1.23 (1.01-1.49) | 1.20 (0.99-1.46) |
| **Inflammatory arthritis** | 32 (2.81) | 4635 (2.08) |  | 1.30 (0.92-1.84) | 1.33 (0.91-1.92) | 1.29 (0.89-1.87) |
| Reactive arthritis | 5 (1.81) | 4662 (2.08) |  | 0.82 (0.34-1.96) | 0.77 (0.29-2.04) | 0.74 (0.28-1.99) |
| Rheumatoid arthritis | 23 (3.08) | 4644 (2.08) |  | 1.43 (0.95-2.15) | 1.45 (0.94-2.25) | 1.40 (0.90-2.18) |
| **Vasculitis autoimmune diseases** | 13 (3.35) | 4654 (2.08) |  | 1.58 (0.92-2.72) | 1.79 (1.04-3.08) | 1.69 (0.98-2.91) |
| Henoch-Schonlein purpura | 8 (2.74) | 4659 (2.08) |  | 1.29 (0.64-2.58) | 1.47 (0.73-2.93) | 1.43 (0.71-2.86) |
| **Connective tissue autoimmune diseases** | 23 (4.93) | 4644 (2.07) |  | 2.34 (1.55-3.52) | 2.20 (1.40-3.46) | 2.04 (1.30-3.21) |
| Systemic lupus erythematosus | 11 (5.16) | 4656 (2.08) |  | 2.43 (1.34-4.38) | 2.56 (1.38-4.76) | 2.27 (1.22-4.22) |
| Systematic sclerosis | 6 (7.90) | 4661 (2.08) |  | 3.79 (1.70-8.44) | 2.18 (0.70-6.75) | 2.05 (0.66-6.35) |
| **Skin autoimmune diseases** | 30 (2.03) | 4637 (2.08) |  | 0.90 (0.63-1.29) | 0.89 (0.60-1.32) | 0.88 (0.60-1.31) |
| Psoriasis vulgaris | 27 (2.11) | 4640 (2.08) |  | 0.94 (0.65-1.38) | 0.91 (0.60-1.38) | 0.90 (0.59-1.36) |
| **Blood autoimmune diseases** | 6 (2.80) | 4661 (2.08) |  | 1.29 (0.58-2.88) | 0.99 (0.37-2.65) | 0.95 (0.36-2.54) |
| **Nervous autoimmune diseases** | 14 (2.95) | 4653 (2.08) |  | 1.37 (0.81-2.32) | 1.62 (0.96-2.73) | 1.59 (0.94-2.69) |
| Multiple sclerosis | 12 (3.60) | 4655 (2.08) |  | 1.67 (0.95-2.94) | 1.98 (1.13-3.50) | 1.97 (1.12-3.47) |
| **Gastrointestinal autoimmune diseases** | 60 (2.12) | 4607 (2.08) |  | 0.98 (0.76-1.26) | 1.09 (0.84-1.42) | 1.07 (0.83-1.39) |
| Crohn disease | 20 (2.39) | 4647 (2.08) |  | 1.13 (0.73-1.75) | 1.28 (0.83-1.99) | 1.26 (0.81-1.96) |
| Ulcerative colitis | 22 (1.96) | 4645 (2.08) |  | 0.92 (0.60-1.39) | 1.02 (0.67-1.57) | 1.01 (0.66-1.55) |
| Celiac disease | 22 (1.95) | 4645 (2.08) |  | 0.88 (0.58-1.33) | 0.96 (0.62-1.48) | 0.93 (0.60-1.45) |
| **Other autoimmune diseases** | 15 (2.77) | 4652 (2.08) |  | 1.33 (0.80-2.20) | 1.46 (0.88-2.42) | 1.37 (0.82-2.27) |
| IgA nephropathy | 11 (3.09) | 4656 (2.08) |  | 1.50 (0.83-2.71) | 1.63 (0.90-2.94) | 1.55 (0.86-2.80) |

CI: Confidence interval; HR: Hazard ratio; PY: Person-year.

Only autoimmune diseases with ≥ 5 deaths occurring in the exposed group were included in our analyses.

Models 1 were adjusted for offspring age (as the underlying time scale in the Cox regression models); Models 2 were additionally adjusted for offspring’s calendar year of birth, parity, maternal age, country of birth, education, and marital status at the time of birth; Models 3 were adjusted for factors in model 2 and for maternal smoking in early pregnancy, maternal cardiovascular diseases and diabetes before childbirth.

Supplementary Table S4 Hazard ratios and 95% confidence intervals for mortality caused by certain conditions originating in the perinatal period in offspring according to maternal autoimmune diseases during pregnancy

| Exposure | Events (per 10,000 PYs) | | HR (95% CI) |
| --- | --- | --- | --- |
|  | Exposed | Non-exposed |  |
| **Any autoimmune diseases** | 144 (1.13) | 1559 (0.74) | 1.43 (1.18-1.72) |
| **Endocrine autoimmune diseases** | 75 (1.25) | 1628 (0.74) | 1.57 (1.21-2.04) |
| Type 1 diabetes | 28 (1.85) | 1675 (0.75) | 3.49 (1.94-6.30) |
| Autoimmune thyroiditis | 52 (1.11) | 1651 (0.75) | 1.38 (1.03-1.84) |
| **Inflammatory arthritis** | 14 (1.23) | 1689 (0.76) | 1.57 (0.91-2.71) |
| Rheumatoid arthritis | 11 (1.47) | 1692 (0.76) | 1.84 (0.99-3.43) |
| **Connective tissue autoimmune diseases** | 18 (3.85) | 1685 (0.75) | 3.98 (2.35-6.75) |
| Systemic lupus erythematosus | 9 (4.22) | 1694 (0.75) | 4.70 (2.34-9.45) |
| Systematic sclerosis | 5 (6.57) | 1698 (0.76) | 3.59 (0.9-14.38) |
| **Skin autoimmune diseases** | 11 (0.74) | 1692 (0.76) | 0.74 (0.37-1.49) |
| Psoriasis vulgaris | 9 (0.70) | 1694 (0.76) | 0.64 (0.29-1.43) |
| **Nervous autoimmune diseases** | 6 (1.26) | 1697 (0.76) | 1.78 (0.80-3.97) |
| **Gastrointestinal autoimmune diseases** | 23 (0.81) | 1680 (0.76) | 1.04 (0.67-1.60) |
| Crohn disease | 8 (0.95) | 1695 (0.76) | 1.33 (0.67-2.67) |
| Ulcerative colitis | 11 (0.98) | 1692 (0.76) | 1.26 (0.68-2.35) |
| Celiac disease | 6 (0.53) | 1697 (0.76) | 0.61 (0.25-1.47) |
| **Other autoimmune diseases** | 6 (1.10) | 1697 (0.76) | 1.46 (0.66-3.27) |
| IgA nephropathy | 5 (1.40) | 1698 (0.76) | 1.91 (0.79-4.60) |

CI: Confidence interval; HR: Hazard ratio; PY: Person-year.

Only autoimmune diseases with ≥ 5 deaths occurring in the exposed group were included in our analyses.

Analyses were adjusted for offspring’s calendar year of birth, parity, maternal age, country of birth, education, and marital status at the time of birth, maternal smoking in early pregnancy, maternal cardiovascular diseases and diabetes before childbirth.

Supplementary Table S5 Hazard ratios and 95% confidence intervals for congenital malformations and chromosomal abnormalities mortality in offspring according to maternal autoimmune diseases during pregnancy

| Exposure | Events (per 10,000 PYs) | | HR (95% CI) |
| --- | --- | --- | --- |
|  | Exposed | Non-exposed |  |
| **Any autoimmune diseases** | 97 (0.76) | 1252 (0.59) | 1.29 (1.04-1.62) |
| **Endocrine autoimmune diseases** | 51 (0.85) | 1298 (0.59) | 1.37 (1.00-1.87) |
| Type 1 diabetes | 24 (1.59) | 1325 (0.59) | 3.12 (1.67-5.82) |
| Autoimmune thyroiditis | 31 (0.66) | 1318 (0.60) | 1.12 (0.78-1.63) |
| **Inflammatory arthritis** | 6 (0.53) | 1343 (0.60) | 0.98 (0.44-2.18) |
| **Skin autoimmune diseases** | 12 (0.81) | 1337 (0.60) | 1.37 (0.75-2.48) |
| Psoriasis vulgaris | 12 (0.94) | 1337 (0.60) | 1.58 (0.87-2.86) |
| **Nervous autoimmune diseases** | 5 (1.05) | 1344 (0.60) | 2.03 (0.85-4.90) |
| Multiple sclerosis | 5 (1.50) | 1344 (0.60) | 2.95 (1.22-7.10) |
| **Gastrointestinal autoimmune diseases** | 19 (0.67) | 1330 (0.60) | 1.21 (0.76-1.93) |
| Crohn disease | 5 (0.60) | 1344 (0.60) | 1.12 (0.46-2.69) |
| Ulcerative colitis | 8 (0.71) | 1341 (0.60) | 1.37 (0.68-2.74) |
| Celiac disease | 7 (0.62) | 1342 (0.60) | 1.02 (0.46-2.27) |

CI: Confidence interval; HR: Hazard ratio; PY: Person-year.

Only autoimmune diseases with ≥ 5 deaths occurring in the exposed group were included in our analyses.

Analyses were adjusted for age, offspring’s calendar year of birth, parity, maternal age, country of birth, education, and marital status at the time of birth, maternal smoking in early pregnancy, maternal cardiovascular diseases and diabetes before childbirth.

Supplementary Table S6 Hazard ratios and 95% confidence intervals for death due to other natural cause in offspring according to maternal any autoimmune diseases during pregnancy

| Cause of death | Events (per 10,000 PYs) | | HR (95% CI) |
| --- | --- | --- | --- |
|  | Exposed | Non-exposed |  |
| Infectious and parasitic diseases | 10 (0.08) | 151 (0.07) | 1.09 (0.54-2.19) |
| Malignant neoplasms | 28 (0.22) | 493 (0.23) | 0.98 (0.65-1.45) |
| Endocrine, nutritional and metabolic diseases | 14 (0.11) | 211 (0.10) | 1.15 (0.65-2.02) |
| Diseases of the nervous system and the sense organs | 19 (0.15) | 332 (0.16) | 0.91 (0.54-1.53) |
| Diseases of the circulatory system | 7 (0.05) | 142 (0.07) | 0.85 (0.37-1.97) |
| Diseases of the respiratory system | 13 (0.10) | 126 (0.06) | 1.82 (1.00-3.35) |

CI: Confidence interval; HR: Hazard ratio; PY: Person-year.

Deaths with ≥ 5 events occurring in the exposed group were presented.

Analyses were adjusted for offspring’s calendar year of birth, parity, maternal age, country of birth, education, and marital status at the time of birth, maternal smoking in early pregnancy, maternal cardiovascular diseases and diabetes before childbirth.

Supplementary Table S7 Hazard ratios and 95% confidence intervals for death due to unnatural causes in offspring according to maternal autoimmune diseases during pregnancy

| Exposure | Events (per 10,000 PYs) | | HR (95% CI) |
| --- | --- | --- | --- |
|  | Exposed | Non-exposed |  |
| **Any autoimmune diseases** | 44 (0.34) | 750 (0.35) | 1.16 (0.83-1.62) |
| **Endocrine autoimmune diseases** | 20 (0.33) | 774 (0.35) | 1.28 (0.78-2.09) |
| Type 1 diabetes | 9 (0.59) | 785 (0.35) | 1.56 (0.64-3.78) |
| Autoimmune thyroiditis | 12 (0.26) | 782 (0.36) | 1.20 (0.68-2.14) |
| **Gastrointestinal autoimmune diseases** | 15 (0.53) | 779 (0.35) | 1.67 (0.97-2.90) |
| Ulcerative colitis | 6 (0.53) | 788 (0.35) | 1.65 (0.68-3.97) |
| Celiac disease | 9 (0.80) | 785 (0.35) | 2.72 (1.35-5.46) |

CI: Confidence interval; HR: Hazard ratio; PY: Person-year.

Only autoimmune diseases with ≥ 5 deaths occurring in the exposed group were included in our analyses.

Analyses were adjusted for offspring’s calendar year of birth, parity, maternal age, country of birth, education, and marital status at the time of birth, maternal smoking in early pregnancy, maternal cardiovascular diseases and diabetes before childbirth.

Supplementary Table S8 Hazard ratios and 95% confidence intervals for mortality due to cause-specific mortality in offspring according to paternal autoimmune diseases during the partner’s pregnancy

| Cause of death | Events (per 10,000 PYs) | | HR (95% CI) | HR ratio (95% CI)* | P value |
| --- | --- | --- | --- | --- | --- |
|  | Exposed | Non-exposed |  |  |  |
| Natural causes | 115 (1.68) | 3073 (1.83) | 0.88 (0.72-1.08) | 1.43 (1.13-1.81) | 0.003 |
| Congenital malformations and chromosomal abnormalities | 30 (0.44) | 866 (0.51) | 0.75 (0.50-1.13) | 1.72 (1.08-2.74) | 0.02 |
| Certain conditions originating in the perinatal period | 38 (0.55) | 1081 (0.64) | 0.87 (0.62-1.23) | 1.64 (1.11-2.43) | 0.01 |
| Unnatural causes | 26 (0.38) | 584 (0.35) | 1.31 (0.86-1.98) | 0.89 (0.52-1.51) | 0.66 |

CI: Confidence interval; HR: Hazard ratio; PY: Person-year.

Only autoimmune diseases with ≥ 5 deaths occurring in the exposed group were included in our analyses.

Analyses were adjusted foroffspring’s calendar year of birth, paternal age, country of birth, education, and marital status at the time of birth, paternal cardiovascular diseases and diabetes before childbirth.

* The maternal-to-paternal association ratio was defined as the adjusted hazard ratio for maternal autoimmune disease divided by the corresponding adjusted hazard ratio for paternal autoimmune disease. The 95% confidence intervals were calculated using the Wald method.

Supplementary Table S9 Hazard ratios and 95% confidence intervals for cause-specific mortality in offspring according to maternal autoimmune disease during pregnancy in cousin analyses

| Cause of death | Events (per 10,000 PYs) | | HR (95% CI) |
| --- | --- | --- | --- |
|  | Exposed | Non-exposed |  |
| Natural causes | 183 (2.71) | 1940 (1.79) | 0.99 (0.74-1.31) |
| Congenital malformations and chromosomal abnormalities | 55 (0.81) | 559 (0.51) | 0.99 (0.58-1.70) |
| Certain conditions originating in the perinatal period | 71 (1.05) | 701 (0.65) | 1.18 (0.73-1.90) |
| Unnatural causes | 28 (0.41) | 354 (0.33) | 1.66 (0.72-3.81) |

CI: Confidence interval; HR: Hazard ratio; PY: Person-year.

Only autoimmune diseases with ≥ 5 deaths occurring in the exposed group were included in our analyses.

Analyses were adjusted foroffspring’s calendar year of birth, parity, maternal age, country of birth, education, and marital status at the time of birth, maternal smoking in early pregnancy, maternal cardiovascular diseases and diabetes before childbirth.

Supplementary Table S10 Hazard ratios and 95% confidence intervals for all-cause mortality in offspring according to maternal autoimmune diseases by sex

| Exposure | Male (n = 748,328) | |  | Female (n = 707,317) | |  |
| --- | --- | --- | --- | --- | --- | --- |
|  | Events (per 10,000 PYs) | | HR (95% CI) | Events (per 10,000 PYs) | | HR (95% CI) |
|  | Exposed | Non-exposed |  | Exposed | Non-exposed |  |
| **Any autoimmune disease** | 241 (3.70) | 3210 (2.95) | 1.28 (1.11-1.48) | 185 (2.98) | 2518 (2.45) | 1.14 (0.97-1.35) |
| **Endocrine autoimmune diseases** | 124 (4.04) | 3327 (2.97) | 1.45 (1.19-1.77) | 96 (3.30) | 2607 (2.46) | 1.16 (0.92-1.47) |
| Type 1 diabetes | 41 (5.34) | 3410 (2.98) | 2.23 (1.43-3.47) | 43 (5.85) | 2660 (2.46) | 1.78 (1.16-2.74) |
| Autoimmune thyroiditis | 89 (3.73) | 3362 (2.98) | 1.35 (1.08-1.68) | 59 (2.60) | 2644 (2.48) | 1.00 (0.76-1.33) |
| **Inflammatory arthritis** | 23 (4.02) | 3428 (2.99) | 1.31 (0.84-2.03) | 17 (3.02) | 2686 (2.48) | 1.10 (0.65-1.86) |
| Rheumatoid arthritis | 19 (5.06) | 3432 (2.99) | 1.60 (0.98-2.61) | 12 (3.23) | 2691 (2.48) | 1.19 (0.64-2.22) |
| **Connective tissue autoimmune diseases** | 18 (7.66) | 3433 (2.99) | 2.14 (1.27-3.63) | 7 (3.02) | 2696 (2.48) | 1.29 (0.61-2.70) |
| **Skin autoimmune diseases** | 19 (2.49) | 3432 (3.0) | 0.78 (0.48-1.28) | 20 (2.80) | 2683 (2.48) | 1.12 (0.71-1.79) |
| Psoriasis vulgaris | 15 (2.27) | 3436 (3.0) | 0.67 (0.38-1.19) | 20 (3.23) | 2683 (2.48) | 1.30 (0.82-2.07) |
| **Nervous system autoimmune diseases** | 8 (3.24) | 3443 (2.99) | 1.28 (0.64-2.56) | 8 (3.52) | 2695 (2.48) | 1.62 (0.81-3.25) |
| Multiple sclerosis | 6 (3.50) | 3445 (2.99) | 1.42 (0.64-3.16) | 7 (4.34) | 2696 (2.48) | 2.03 (0.97-4.26) |
| **Gastrointestinal autoimmune diseases** | 47 (3.28) | 3404 (2.99) | 1.10 (0.81-1.50) | 33 (2.37) | 2670 (2.48) | 1.00 (0.70-1.42) |
| Crohn disease | 14 (3.27) | 3437 (2.99) | 1.12 (0.65-1.93) | 9 (2.20) | 2694 (2.48) | 0.99 (0.52-1.91) |
| Ulcerative colitis | 17 (3.0) | 3434 (2.99) | 0.96 (0.57-1.62) | 13 (2.35) | 2690 (2.48) | 1.07 (0.62-1.84) |
| Celiac disease | 21 (3.67) | 3430 (2.99) | 1.23 (0.79-1.94) | 12 (2.17) | 2691 (2.48) | 0.79 (0.42-1.47) |
| **Other autoimmune diseases** | 13 (4.65) | 3438 (2.99) | 1.63 (0.94-2.81) | 5 (1.90) | 2698 (2.48) | 0.80 (0.33-1.92) |

CI: Confidence interval; HR: Hazard ratio; PY: Person-year.

Only autoimmune diseases with ≥ 5 deaths occurring in the exposed group were included in our analyses.

Analyses were adjusted for offspring’s calendar year of birth, parity, maternal country of birth, age, education, and marital status at the time of birth, maternal smoking in early pregnancy, maternal cardiovascular diseases and diabetes before childbirth.

Supplementary Table S11 Hazard ratios and 95% confidence intervals for all-cause mortality in offspring according to maternal autoimmune diseases by small for gestational age

| Exposure | Small for gestational age (n = 148,424) | | | Not small for gestational age (n = 1,303,833) | | |
| --- | --- | --- | --- | --- | --- | --- |
|  | Events (per 10,000 PYs) | | HR (95% CI) | Events (per 10,000 PYs) | | HR (95% CI) |
|  | Exposed | Non-exposed |  | Exposed | Non-exposed |  |
| **Any autoimmune disease** | 125 (9.42) | 1582 (7.48) | 1.21 (0.99-1.46) | 281 (2.47) | 3946 (2.08) | 1.15 (1.00-1.31) |
| **Endocrine autoimmune diseases** | 54 (9.28) | 1653 (7.55) | 1.12 (0.84-1.50) | 154 (2.86) | 4073 (2.08) | 1.32 (1.10-1.58) |
| Type 1 diabetes | 14 (19.22) | 1693 (7.56) | 1.90 (0.97-3.75) | 67 (4.70) | 4160 (2.08) | 2.07 (1.45-2.95) |
| Autoimmune thyroiditis | 42 (8.13) | 1665 (7.59) | 1.04 (0.75-1.44) | 97 (2.35) | 4130 (2.10) | 1.16 (0.94-1.44) |
| **Inflammatory arthritis** | 11 (7.95) | 1696 (7.60) | 0.89 (0.46-1.71) | 27 (2.71) | 4200 (2.10) | 1.23 (0.82-1.86) |
| Rheumatoid arthritis | 10 (10.65) | 1697 (7.59) | 1.14 (0.57-2.28) | 19 (2.92) | 4208 (2.10) | 1.31 (0.80-2.14) |
| **Vasculitis** | 9 (22.0) | 1698 (7.57) | 3.02 (1.57-5.83) | 6 (1.73) | 4221 (2.10) | 0.72 (0.30-1.73) |
| **Connective tissue autoimmune diseases** | 7 (9.79) | 1700 (7.59) | 1.02 (0.45-2.27) | 15 (3.82) | 4212 (2.10) | 1.60 (0.91-2.83) |
| **Skin autoimmune diseases** | 12 (7.75) | 1695 (7.60) | 0.99 (0.55-1.79) | 22 (1.67) | 4205 (2.10) | 0.72 (0.46-1.15) |
| Psoriasis vulgaris | 11 (8.14) | 1696 (7.60) | 1.01 (0.54-1.88) | 19 (1.66) | 4208 (2.10) | 0.70 (0.42-1.16) |
| **Gastrointestinal autoimmune diseases** | 26 (9.0) | 1681 (7.58) | 1.22 (0.82-1.81) | 53 (2.10) | 4174 (2.10) | 0.99 (0.74-1.32) |
| Crohn disease | 9 (9.98) | 1698 (7.59) | 1.21 (0.60-2.43) | 13 (1.75) | 4214 (2.10) | 0.93 (0.54-1.60) |
| Ulcerative colitis | 11 (9.87) | 1696 (7.59) | 1.31 (0.70-2.44) | 18 (1.79) | 4209 (2.10) | 0.87 (0.53-1.43) |
| Celiac disease | 8 (6.86) | 1699 (7.60) | 0.94 (0.47-1.89) | 25 (2.48) | 4202 (2.10) | 1.09 (0.71-1.67) |
| **Other autoimmune diseases** | 10 (15.07) | 1697 (7.58) | 1.89 (1.01-3.54) | 8 (1.69) | 4219 (2.10) | 0.85 (0.43-1.70) |

CI: Confidence interval; HR: Hazard ratio; PY: Person-year.

Only autoimmune diseases with ≥ 5 deaths occurring in the exposed group were included in our analyses.

Analyses were adjusted for offspring’s calendar year of birth, parity, maternal country of birth, age, education, and marital status at the time of birth, maternal smoking in early pregnancy, maternal cardiovascular diseases and diabetes before childbirth.

Supplementary Table S12 Hazard ratios and 95% confidence intervals for mortality in offspring according to maternal autoimmune diseases during pregnancy in individuals who had data on maternal family history of diabetes (n= 1,210,478)

| Outcome | Exposure | Events (per 10,000 PYs) | |  | HR (95% CI) | |
| --- | --- | --- | --- | --- | --- | --- |
|  |  | Exposed | Non-exposed |  | Model 1 | Model 2 |
| **All cause of death** | **Any AID** | 371 (3.28) | 4552 (2.55) |  | 1.25 (1.11-1.40) | 1.25 (1.11-1.40) |
|  | **Endocrine autoimmune diseases** | 182 (3.66) | 4741 (2.57) |  | 1.41 (1.19-1.67) | 1.41 (1.19-1.67) |
|  | Type 1 diabetes | 73 (5.68) | 4850 (2.58) |  | 2.34 (1.61-3.38) | 2.34 (1.61-3.38) |
|  | Autoimmune thyroiditis | 118 (3.06) | 4805 (2.59) |  | 1.24 (1.02-1.50) | 1.24 (1.02-1.50) |
|  | **Inflammatory arthritis** | 38 (3.55) | 4885 (2.59) |  | 1.27 (0.90-1.80) | 1.27 (0.90-1.80) |
|  | Reactive arthritis | 5 (1.94) | 4918 (2.60) |  | 0.64 (0.24-1.70) | 0.64 (0.24-1.70) |
|  | Rheumatoid arthritis | 29 (4.10) | 4894 (2.59) |  | 1.46 (0.98-2.16) | 1.46 (0.98-2.16) |
|  | **Vasculitis** | 15 (4.03) | 4908 (2.59) |  | 1.45 (0.86-2.45) | 1.45 (0.86-2.45) |
|  | Henoch-Schonlein purpura | 10 (3.53) | 4913 (2.60) |  | 1.27 (0.66-2.43) | 1.27 (0.66-2.43) |
|  | **Connective tissue autoimmune diseases** | 23 (5.40) | 4900 (2.59) |  | 1.77 (1.12-2.77) | 1.77 (1.12-2.77) |
|  | Systemic lupus erythematosus | 11 (5.68) | 4912 (2.59) |  | 1.99 (1.07-3.71) | 1.99 (1.07-3.71) |
|  | Sjögren’s disease | 6 (8.18) | 4917 (2.60) |  | 1.63 (0.52-5.04) | 1.62 (0.52-5.04) |
|  | **Skin autoimmune diseases** | 36 (2.64) | 4887 (2.60) |  | 0.94 (0.66-1.34) | 0.94 (0.66-1.34) |
|  | Psoriasis vulgaris | 33 (2.77) | 4890 (2.60) |  | 0.97 (0.67-1.41) | 0.97 (0.67-1.41) |
|  | **Blood autoimmune diseases** | 6 (3.13) | 4917 (2.60) |  | 1.06 (0.44-2.56) | 1.06 (0.44-2.56) |
|  | **Nervous system autoimmune diseases** | 14 (3.20) | 4909 (2.60) |  | 1.38 (0.81-2.33) | 1.38 (0.81-2.33) |
|  | Multiple sclerosis | 11 (3.56) | 4912 (2.60) |  | 1.57 (0.87-2.84) | 1.57 (0.87-2.84) |
|  | **Gastrointestinal autoimmune diseases** | 73 (2.70) | 4850 (2.60) |  | 1.01 (0.79-1.29) | 1.01 (0.79-1.29) |
|  | Crohn disease | 19 (2.39) | 4904 (2.60) |  | 0.93 (0.59-1.48) | 0.93 (0.59-1.48) |
|  | Ulcerative colitis | 28 (2.64) | 4895 (2.60) |  | 1.00 (0.68-1.48) | 1.00 (0.68-1.48) |
|  | Celiac disease | 31 (2.83) | 4892 (2.60) |  | 1.00 (0.68-1.45) | 1.00 (0.68-1.45) |
|  | **Other autoimmune diseases** | 16 (3.22) | 4907 (2.60) |  | 1.25 (0.77-2.05) | 1.25 (0.77-2.05) |
|  | IgA nephropathy | 12 (3.67) | 4911 (2.60) |  | 1.42 (0.81-2.51) | 1.43 (0.81-2.51) |
| **Natural cause of death** | **Any AID** | 290 (2.56) | 3412 (1.91) |  | 1.27 (1.11-1.44) | 1.27 (1.11-1.44) |
|  | **Endocrine autoimmune diseases** | 144 (2.89) | 3558 (1.93) |  | 1.41 (1.17-1.70) | 1.41 (1.17-1.70) |
|  | Type 1 diabetes | 58 (4.51) | 3644 (1.93) |  | 2.61 (1.71-3.98) | 2.61 (1.72-3.98) |
|  | Autoimmune thyroiditis | 93 (2.41) | 3609 (1.94) |  | 1.22 (0.98-1.51) | 1.21 (0.98-1.51) |
|  | **Inflammatory arthritis** | 31 (2.89) | 3671 (1.95) |  | 1.35 (0.92-1.97) | 1.35 (0.92-1.97) |
|  | Reactive arthritis | 5 (1.94) | 3697 (1.95) |  | 0.82 (0.31-2.18) | 0.82 (0.31-2.18) |
|  | Rheumatoid arthritis | 22 (3.11) | 3680 (1.95) |  | 1.44 (0.91-2.25) | 1.44 (0.91-2.25) |
|  | **Vasculitis** | 13 (3.49) | 3689 (1.95) |  | 1.77 (1.03-3.05) | 1.77 (1.03-3.05) |
|  | Henoch-Schonlein purpura | 8 (2.82) | 3694 (1.95) |  | 1.49 (0.75-2.99) | 1.49 (0.75-2.99) |
|  | **Connective tissue autoimmune diseases** | 21 (4.93) | 3681 (1.95) |  | 2.03 (1.26-3.27) | 2.03 (1.26-3.27) |
|  | Systemic lupus erythematosus | 11 (5.68) | 3691 (1.95) |  | 2.52 (1.36-4.70) | 2.53 (1.36-4.70) |
|  | Sjögren’s disease | 6 (8.18) | 3696 (1.95) |  | 2.12 (0.68-6.58) | 2.12 (0.68-6.57) |
|  | **Skin autoimmune diseases** | 27 (1.98) | 3675 (1.95) |  | 0.87 (0.57-1.32) | 0.87 (0.57-1.32) |
|  | Psoriasis vulgaris | 25 (2.10) | 3677 (1.95) |  | 0.90 (0.58-1.40) | 0.90 (0.58-1.40) |
|  | **Blood autoimmune diseases** | 5 (2.61) | 3697 (1.95) |  | 1.11 (0.42-2.96) | 1.11 (0.42-2.96) |
|  | **Nervous system autoimmune diseases** | 13 (2.97) | 3689 (1.95) |  | 1.63 (0.94-2.81) | 1.63 (0.94-2.81) |
|  | Multiple sclerosis | 11 (3.56) | 3691 (1.95) |  | 1.98 (1.10-3.58) | 1.98 (1.10-3.58) |
|  | **Gastrointestinal autoimmune diseases** | 53 (1.96) | 3649 (1.95) |  | 1.00 (0.75-1.32) | 1.00 (0.75-1.32) |
|  | Crohn disease | 16 (2.01) | 3686 (1.95) |  | 1.08 (0.66-1.77) | 1.08 (0.66-1.77) |
|  | Ulcerative colitis | 20 (1.88) | 3682 (1.95) |  | 0.98 (0.62-1.54) | 0.98 (0.62-1.54) |
|  | Celiac disease | 20 (1.82) | 3682 (1.95) |  | 0.87 (0.55-1.39) | 0.87 (0.55-1.39) |
|  | **Other autoimmune diseases** | 14 (2.82) | 3688 (1.95) |  | 1.43 (0.84-2.41) | 1.43 (0.84-2.41) |
|  | IgA nephropathy | 11 (3.36) | 3691 (1.95) |  | 1.72 (0.95-3.11) | 1.72 (0.95-3.11) |
| **Unnatural cause of death** | **Any AID** | 40 (0.35) | 616 (0.34) |  | 1.27 (0.90-1.79) | 1.27 (0.90-1.80) |
|  | **Endocrine autoimmune diseases** | 18 (0.36) | 638 (0.34) |  | 1.55 (0.92-2.61) | 1.55 (0.92-2.62) |
|  | Type 1 diabetes | 8 (0.62) | 648 (0.34) |  | 2.38 (0.80-7.06) | 2.38 (0.80-7.06) |
|  | Autoimmune thyroiditis | 11 (0.28) | 645 (0.35) |  | 1.40 (0.77-2.55) | 1.40 (0.77-2.56) |
|  | **Gastrointestinal autoimmune diseases** | 15 (0.55) | 641 (0.34) |  | 1.77 (1.02-3.07) | 1.77 (1.02-3.07) |
|  | Ulcerative colitis | 6 (0.56) | 650 (0.34) |  | 1.77 (0.73-4.26) | 1.77 (0.73-4.26) |
|  | Celiac disease | 9 (0.82) | 647 (0.34) |  | 2.85 (1.42-5.74) | 2.85 (1.42-5.74) |

AID: Autoimmune disease; CI: Confidence interval; HR: Hazard ratio; PY: Person-year.

Only autoimmune diseases with ≥ 5 deaths occurring in the exposed group were included in our analyses.

Models 1 were adjusted for offspring’s calendar year of birth, maternal country of birth, parity, age, education, and marital status at the time of birth, maternal smoking in early pregnancy, maternal cardiovascular diseases and diabetes before childbirth, Models 2 were additionally adjusted for maternal family history of diabetes.

Supplementary Table S13 Hazard ratios and 95% confidence intervals for mortality in offspring according to maternal autoimmune diseases during pregnancy in individuals who had data on paternal autoimmune diseases before childbirth (n=1,122,754)

| Outcome | Exposure | Events (per 10,000 PYs) | |  | HR (95% CI) | |
| --- | --- | --- | --- | --- | --- | --- |
|  |  | Exposed | Non-exposed |  | Model 1 | Model 2 |
| **All cause of death** | **Any AID** | 335 (3.27) | 3932 (2.39) |  | 1.36 (1.20-1.53) | 1.36 (1.20-1.53) |
|  | **Endocrine autoimmune diseases** | 169 (3.74) | 4098 (2.40) |  | 1.55 (1.30-1.85) | 1.55 (1.30-1.85) |
|  | Type 1 diabetes | 64 (5.39) | 4203 (2.42) |  | 2.61 (1.75-3.90) | 2.61 (1.75-3.90) |
|  | Autoimmune thyroiditis | 112 (3.23) | 4155 (2.42) |  | 1.36 (1.11-1.66) | 1.36 (1.11-1.66) |
|  | **Inflammatory arthritis** | 33 (3.43) | 4234 (2.43) |  | 1.35 (0.93-1.94) | 1.35 (0.93-1.94) |
|  | Reactive arthritis | 5 (2.16) | 4262 (2.44) |  | 0.77 (0.29-2.06) | 0.77 (0.29-2.06) |
|  | Rheumatoid arthritis | 25 (3.93) | 4242 (2.43) |  | 1.54 (1.01-2.34) | 1.54 (1.01-2.34) |
|  | **Vasculitis** | 13 (3.88) | 4254 (2.44) |  | 1.50 (0.85-2.65) | 1.50 (0.85-2.65) |
|  | Henoch-Schonlein purpura | 9 (3.54) | 4258 (2.44) |  | 1.36 (0.68-2.73) | 1.36 (0.68-2.73) |
|  | **Connective tissue autoimmune diseases** | 17 (4.41) | 4250 (2.43) |  | 1.76 (1.08-2.88) | 1.76 (1.08-2.88) |
|  | Systemic lupus erythematosus | 9 (5.14) | 4258 (2.44) |  | 2.11 (1.09-4.06) | 2.11 (1.09-4.06) |
|  | **Skin autoimmune diseases** | 31 (2.48) | 4236 (2.44) |  | 0.98 (0.67-1.43) | 0.98 (0.67-1.43) |
|  | Psoriasis vulgaris | 29 (2.66) | 4238 (2.44) |  | 1.03 (0.70-1.53) | 1.03 (0.70-1.53) |
|  | **Nervous system autoimmune diseases** | 15 (3.90) | 4252 (2.44) |  | 1.79 (1.08-2.98) | 1.79 (1.08-2.98) |
|  | Multiple sclerosis | 12 (4.44) | 4255 (2.44) |  | 2.09 (1.19-3.69) | 2.09 (1.19-3.69) |
|  | **Gastrointestinal autoimmune diseases** | 67 (2.72) | 4200 (2.43) |  | 1.11 (0.86-1.43) | 1.11 (0.86-1.43) |
|  | Crohn disease | 16 (2.23) | 4251 (2.44) |  | 0.92 (0.56-1.53) | 0.92 (0.56-1.53) |
|  | Ulcerative colitis | 26 (2.69) | 4241 (2.44) |  | 1.08 (0.72-1.63) | 1.08 (0.72-1.63) |
|  | Celiac disease | 29 (2.90) | 4238 (2.44) |  | 1.15 (0.78-1.69) | 1.15 (0.78-1.69) |
|  | **Other autoimmune diseases** | 12 (2.74) | 4255 (2.44) |  | 1.14 (0.65-2.02) | 1.14 (0.65-2.02) |
|  | IgA nephropathy | 7 (2.40) | 4260 (2.44) |  | 1.00 (0.48-2.10) | 1.00 (0.48-2.10) |
| **Natural cause of death** | **Any AID** | 260 (2.54) | 2928 (1.78) |  | 1.38 (1.20-1.58) | 1.38 (1.20-1.58) |
|  | **Endocrine autoimmune diseases** | 133 (2.94) | 3055 (1.79) |  | 1.54 (1.26-1.87) | 1.54 (1.26-1.87) |
|  | Type 1 diabetes | 51 (4.29) | 3137 (1.80) |  | 2.82 (1.80-4.43) | 2.82 (1.80-4.43) |
|  | Autoimmune thyroiditis | 87 (2.50) | 3101 (1.81) |  | 1.32 (1.05-1.65) | 1.32 (1.05-1.65) |
|  | **Inflammatory arthritis** | 28 (2.91) | 3160 (1.81) |  | 1.51 (1.02-2.24) | 1.51 (1.02-2.24) |
|  | Reactive arthritis | 5 (2.16) | 3183 (1.82) |  | 1.00 (0.37-2.66) | 1.00 (0.37-2.66) |
|  | Rheumatoid arthritis | 20 (3.14) | 3168 (1.82) |  | 1.64 (1.03-2.61) | 1.64 (1.03-2.61) |
|  | **Vasculitis** | 11 (3.29) | 3177 (1.82) |  | 1.83 (1.01-3.31) | 1.83 (1.01-3.31) |
|  | Henoch-Schonlein purpura | 7 (2.75) | 3181 (1.82) |  | 1.60 (0.76-3.35) | 1.60 (0.76-3.35) |
|  | **Connective tissue autoimmune diseases** | 16 (4.15) | 3172 (1.82) |  | 2.15 (1.29-3.58) | 2.15 (1.29-3.58) |
|  | Systemic lupus erythematosus | 9 (5.14) | 3179 (1.82) |  | 2.72 (1.41-5.23) | 2.72 (1.41-5.24) |
|  | **Skin autoimmune diseases** | 22 (1.76) | 3166 (1.82) |  | 0.84 (0.53-1.34) | 0.84 (0.53-1.34) |
|  | Psoriasis vulgaris | 21 (1.92) | 3167 (1.82) |  | 0.91 (0.57-1.47) | 0.91 (0.57-1.47) |
|  | **Nervous system autoimmune diseases** | 13 (3.38) | 3175 (1.82) |  | 1.99 (1.15-3.43) | 1.99 (1.15-3.43) |
|  | Multiple sclerosis | 11 (4.07) | 3177 (1.82) |  | 2.42 (1.34-4.38) | 2.42 (1.34-4.39) |
|  | **Gastrointestinal autoimmune diseases** | 50 (2.03) | 3138 (1.82) |  | 1.14 (0.86-1.52) | 1.14 (0.86-1.52) |
|  | Crohn disease | 14 (1.95) | 3174 (1.82) |  | 1.14 (0.67-1.92) | 1.14 (0.67-1.92) |
|  | Ulcerative colitis | 19 (1.96) | 3169 (1.82) |  | 1.10 (0.69-1.75) | 1.10 (0.69-1.75) |
|  | Celiac disease | 19 (1.90) | 3169 (1.82) |  | 1.05 (0.66-1.66) | 1.05 (0.66-1.66) |
|  | **Other autoimmune diseases** | 9 (2.05) | 3179 (1.82) |  | 1.13 (0.59-2.17) | 1.13 (0.59-2.17) |
|  | IgA nephropathy | 6 (2.06) | 3182 (1.82) |  | 1.14 (0.51-2.54) | 1.14 (0.51-2.55) |
| **Unnatural cause of death** | **Any AID** | 38 (0.37) | 572 (0.35) |  | 1.40 (0.98-2.00) | 1.40 (0.98-2.00) |
|  | **Endocrine autoimmune diseases** | 17 (0.37) | 593 (0.35) |  | 1.96 (1.17-3.30) | 1.96 (1.17-3.30) |
|  | Type 1 diabetes | 6 (0.50) | 604 (0.35) |  | 4.45 (1.26-15.75) | 4.46 (1.26-15.77) |
|  | Autoimmune thyroiditis | 12 (0.34) | 598 (0.35) |  | 1.76 (0.99-3.14) | 1.76 (0.99-3.14) |
|  | **Gastrointestinal autoimmune diseases** | 12 (0.49) | 598 (0.35) |  | 1.53 (0.82-2.86) | 1.53 (0.82-2.85) |
|  | Ulcerative colitis | 5 (0.51) | 605 (0.35) |  | 1.57 (0.59-4.19) | 1.56 (0.58-4.18) |
|  | Celiac disease | 8 (0.80) | 602 (0.35) |  | 2.81 (1.33-5.93) | 2.80 (1.33-5.92) |

AID: Autoimmune disease; CI: Confidence interval; HR: Hazard ratio; PY: Person-year.

Only autoimmune diseases with ≥ 5 deaths occurring in the exposed group were included in our analyses.

Models 1 were adjusted for offspring’s calendar year of birth, maternal country of birth, parity, age, education, and marital status at the time of birth, maternal smoking in early pregnancy, maternal cardiovascular diseases and diabetes before childbirth, Models 2 were additionally adjusted for paternal autoimmune diseases before childbirth.

Supplementary Table S14 Hazard ratios and 95% confidence intervals for mortality in offspring according to maternal autoimmune diseases during pregnancy in individuals who had data on maternal body mass index in early pregnancy (n=1,321,186)

| Outcome | Exposure | Events (per 10,000 PYs) | |  | HR (95% CI) | |
| --- | --- | --- | --- | --- | --- | --- |
|  |  | Exposed | Non-exposed |  | Model 1 | Model 2 |
| **All cause of death** | **Any AID** | 355 (3.07) | 4766 (2.50) |  | 1.23 (1.10-1.39) | 1.23 (1.09-1.38) |
|  | **Endocrine autoimmune diseases** | 178 (3.26) | 4943 (2.52) |  | 1.27 (1.07-1.50) | 1.25 (1.06-1.48) |
|  | Type 1 diabetes | 67 (4.98) | 5054 (2.52) |  | 2.00 (1.41-2.82) | 2.03 (1.45-2.86) |
|  | Autoimmune thyroiditis | 119 (2.78) | 5002 (2.53) |  | 1.12 (0.92-1.36) | 1.10 (0.90-1.33) |
|  | **Inflammatory arthritis** | 33 (3.22) | 5088 (2.53) |  | 1.26 (0.87-1.81) | 1.26 (0.87-1.81) |
|  | Rheumatoid arthritis | 25 (3.73) | 5096 (2.53) |  | 1.46 (0.96-2.22) | 1.46 (0.96-2.22) |
|  | **Vasculitis** | 14 (4.02) | 5107 (2.53) |  | 1.56 (0.91-2.69) | 1.56 (0.90-2.68) |
|  | Henoch-Schonlein purpura | 9 (3.43) | 5112 (2.53) |  | 1.32 (0.66-2.63) | 1.32 (0.66-2.63) |
|  | **Connective tissue autoimmune diseases** | 19 (4.56) | 5102 (2.53) |  | 1.75 (1.09-2.82) | 1.76 (1.10-2.84) |
|  | Systemic lupus erythematosus | 9 (4.78) | 5112 (2.53) |  | 2.03 (1.06-3.91) | 2.07 (1.07-3.98) |
|  | **Skin autoimmune diseases** | 35 (2.59) | 5086 (2.53) |  | 1.05 (0.74-1.49) | 1.03 (0.73-1.46) |
|  | Psoriasis vulgaris | 31 (2.65) | 5090 (2.53) |  | 1.06 (0.73-1.54) | 1.04 (0.72-1.51) |
|  | **Blood autoimmune diseases** | 6 (3.13) | 5115 (2.53) |  | 1.13 (0.47-2.71) | 1.14 (0.48-2.75) |
|  | **Nervous system autoimmune diseases** | 14 (3.25) | 5107 (2.53) |  | 1.49 (0.88-2.52) | 1.49 (0.88-2.53) |
|  | Multiple sclerosis | 12 (3.94) | 5109 (2.53) |  | 1.85 (1.05-3.26) | 1.85 (1.05-3.27) |
|  | **Gastrointestinal autoimmune diseases** | 67 (2.61) | 5054 (2.53) |  | 1.07 (0.83-1.38) | 1.09 (0.85-1.40) |
|  | Crohn disease | 18 (2.39) | 5103 (2.54) |  | 0.99 (0.62-1.60) | 1.00 (0.62-1.61) |
|  | Ulcerative colitis | 26 (2.56) | 5095 (2.54) |  | 1.07 (0.72-1.60) | 1.08 (0.72-1.61) |
|  | Celiac disease | 28 (2.71) | 5093 (2.53) |  | 1.07 (0.72-1.58) | 1.09 (0.74-1.62) |
|  | **Other autoimmune diseases** | 18 (3.66) | 5103 (2.53) |  | 1.51 (0.95-2.39) | 1.49 (0.94-2.37) |
|  | Sarcoidosis | 5 (3.71) | 5116 (2.53) |  | 1.63 (0.68-3.93) | 1.60 (0.66-3.84) |
|  | IgA nephropathy | 12 (3.71) | 5109 (2.53) |  | 1.52 (0.86-2.68) | 1.50 (0.85-2.65) |
| **Natural cause of death** | **Any AID** | 270 (2.33) | 3584 (1.88) |  | 1.25 (1.10-1.43) | 1.24 (1.09-1.42) |
|  | **Endocrine autoimmune diseases** | 138 (2.53) | 3716 (1.89) |  | 1.29 (1.07-1.56) | 1.27 (1.05-1.54) |
|  | Type 1 diabetes | 52 (3.86) | 3802 (1.89) |  | 2.35 (1.58-3.49) | 2.38 (1.61-3.53) |
|  | Autoimmune thyroiditis | 92 (2.15) | 3762 (1.90) |  | 1.11 (0.89-1.38) | 1.09 (0.87-1.35) |
|  | **Inflammatory arthritis** | 25 (2.44) | 3829 (1.90) |  | 1.31 (0.87-1.97) | 1.31 (0.87-1.97) |
|  | Rheumatoid arthritis | 17 (2.53) | 3837 (1.90) |  | 1.39 (0.85-2.28) | 1.40 (0.85-2.28) |
|  | **Vasculitis** | 12 (3.45) | 3842 (1.90) |  | 1.93 (1.09-3.40) | 1.92 (1.09-3.38) |
|  | Henoch-Schonlein purpura | 7 (2.66) | 3847 (1.91) |  | 1.54 (0.73-3.24) | 1.54 (0.73-3.23) |
|  | **Connective tissue autoimmune diseases** | 18 (4.32) | 3836 (1.90) |  | 2.15 (1.31-3.51) | 2.17 (1.33-3.55) |
|  | Systemic lupus erythematosus | 9 (4.78) | 3845 (1.90) |  | 2.64 (1.37-5.08) | 2.68 (1.39-5.16) |
|  | **Skin autoimmune diseases** | 26 (1.92) | 3828 (1.91) |  | 0.99 (0.65-1.49) | 0.97 (0.64-1.46) |
|  | Psoriasis vulgaris | 23 (1.96) | 3831 (1.91) |  | 0.99 (0.64-1.54) | 0.97 (0.62-1.51) |
|  | **Blood autoimmune diseases** | 5 (2.61) | 3849 (1.91) |  | 1.19 (0.45-3.17) | 1.21 (0.45-3.22) |
|  | **Nervous system autoimmune diseases** | 12 (2.79) | 3842 (1.90) |  | 1.64 (0.93-2.90) | 1.65 (0.93-2.90) |
|  | Multiple sclerosis | 11 (3.61) | 3843 (1.90) |  | 2.16 (1.19-3.90) | 2.16 (1.20-3.91) |
|  | **Gastrointestinal autoimmune diseases** | 47 (1.83) | 3807 (1.91) |  | 1.05 (0.79-1.41) | 1.07 (0.80-1.43) |
|  | Crohn disease | 15 (1.99) | 3839 (1.91) |  | 1.16 (0.70-1.92) | 1.17 (0.70-1.94) |
|  | Ulcerative colitis | 18 (1.77) | 3836 (1.91) |  | 1.05 (0.66-1.66) | 1.06 (0.67-1.68) |
|  | Celiac disease | 17 (1.65) | 3837 (1.91) |  | 0.91 (0.56-1.49) | 0.93 (0.57-1.52) |
|  | **Other autoimmune diseases** | 15 (3.05) | 3839 (1.90) |  | 1.65 (0.99-2.75) | 1.63 (0.98-2.71) |
|  | IgA nephropathy | 11 (3.40) | 3843 (1.90) |  | 1.85 (1.02-3.34) | 1.83 (1.01-3.31) |
| **Unnatural cause of death** | **Any AID** | 41 (0.35) | 642 (0.34) |  | 1.24 (0.87-1.75) | 1.23 (0.87-1.75) |
|  | **Endocrine autoimmune diseases** | 18 (0.33) | 665 (0.34) |  | 1.32 (0.79-2.21) | 1.31 (0.78-2.19) |
|  | Type 1 diabetes | 8 (0.59) | 675 (0.34) |  | 1.62 (0.63-4.16) | 1.66 (0.65-4.23) |
|  | Autoimmune thyroiditis | 11 (0.26) | 672 (0.34) |  | 1.24 (0.68-2.27) | 1.23 (0.67-2.24) |
|  | **Gastrointestinal autoimmune diseases** | 15 (0.58) | 668 (0.33) |  | 1.90 (1.10-3.30) | 1.93 (1.11-3.34) |
|  | Ulcerative colitis | 6 (0.59) | 677 (0.34) |  | 1.87 (0.77-4.51) | 1.88 (0.78-4.54) |
|  | Celiac disease | 9 (0.87) | 674 (0.33) |  | 3.05 (1.51-6.14) | 3.10 (1.54-6.25) |

AID: Autoimmune disease; CI: Confidence interval; HR: Hazard ratio; PY: Person-year.

Only autoimmune diseases with ≥ 5 deaths occurring in the exposed group were included in our analyses.

Models 1 were adjusted for offspring’s calendar year of birth, maternal country of birth, parity, age, education, and marital status at the time of birth, maternal smoking in early pregnancy, maternal cardiovascular diseases and diabetes before childbirth, Models 2 were additionally adjusted for maternal body mass index in early pregnancy.

Supplementary Table S15 Hazard ratios and 95% confidence intervals for mortality in offspring according to maternal autoimmune diseases during pregnancy in individuals who had data on maternal family history of cardiovascular diseases before delivery (n=1,209,500)

| Outcome | Exposure | Events (per 10,000 PYs) | |  | HR (95% CI) | |
| --- | --- | --- | --- | --- | --- | --- |
|  |  | Exposed | Non-exposed |  | Model 1 | Model 2 |
| **All cause of death** | **Any AID** | 371 (3.28) | 4548 (2.55) |  | 1.25 (1.11-1.40) | 1.25 (1.11-1.40) |
|  | **Endocrine autoimmune diseases** | 182 (3.66) | 4737 (2.57) |  | 1.41 (1.19-1.67) | 1.41 (1.19-1.66) |
|  | Type 1 diabetes | 73 (5.68) | 4846 (2.58) |  | 2.33 (1.61-3.38) | 2.33 (1.61-3.38) |
|  | Autoimmune thyroiditis | 118 (3.06) | 4801 (2.59) |  | 1.24 (1.02-1.50) | 1.24 (1.02-1.50) |
|  | **Inflammatory arthritis** | 38 (3.55) | 4881 (2.59) |  | 1.28 (0.91-1.80) | 1.27 (0.90-1.79) |
|  | Reactive arthritis | 5 (1.94) | 4914 (2.60) |  | 0.64 (0.24-1.70) | 0.64 (0.24-1.70) |
|  | Rheumatoid arthritis | 29 (4.10) | 4890 (2.59) |  | 1.46 (0.98-2.16) | 1.46 (0.98-2.16) |
|  | **Vasculitis** | 15 (4.03) | 4904 (2.59) |  | 1.45 (0.86-2.44) | 1.45 (0.86-2.45) |
|  | Henoch-Schonlein purpura | 10 (3.53) | 4909 (2.60) |  | 1.27 (0.66-2.43) | 1.27 (0.66-2.43) |
|  | **Connective tissue autoimmune diseases** | 23 (5.40) | 4896 (2.59) |  | 1.77 (1.13-2.78) | 1.77 (1.12-2.77) |
|  | Systemic lupus erythematosus | 11 (5.68) | 4908 (2.59) |  | 1.99 (1.07-3.70) | 1.99 (1.07-3.70) |
|  | Systematic sclerosis | 6 (8.18) | 4913 (2.60) |  | 1.63 (0.52-5.04) | 1.62 (0.52-5.03) |
|  | **Skin autoimmune diseases** | 36 (2.64) | 4883 (2.60) |  | 0.95 (0.66-1.35) | 0.94 (0.66-1.34) |
|  | Psoriasis vulgaris | 33 (2.78) | 4886 (2.60) |  | 0.97 (0.67-1.41) | 0.97 (0.67-1.41) |
|  | **Blood autoimmune diseases** | 6 (3.13) | 4913 (2.60) |  | 1.06 (0.44-2.56) | 1.06 (0.44-2.56) |
|  | **Nervous system autoimmune diseases** | 14 (3.20) | 4905 (2.60) |  | 1.38 (0.81-2.33) | 1.38 (0.81-2.32) |
|  | Multiple sclerosis | 11 (3.56) | 4908 (2.60) |  | 1.57 (0.87-2.84) | 1.57 (0.87-2.84) |
|  | **Gastrointestinal autoimmune diseases** | 73 (2.70) | 4846 (2.60) |  | 1.01 (0.79-1.29) | 1.01 (0.79-1.29) |
|  | Crohn disease | 19 (2.39) | 4900 (2.60) |  | 0.93 (0.59-1.48) | 0.93 (0.59-1.48) |
|  | Ulcerative colitis | 28 (2.64) | 4891 (2.60) |  | 1.00 (0.68-1.48) | 1.00 (0.68-1.48) |
|  | Celiac disease | 31 (2.83) | 4888 (2.60) |  | 1.00 (0.68-1.45) | 1.00 (0.68-1.45) |
|  | **Other autoimmune diseases** | 16 (3.23) | 4903 (2.60) |  | 1.25 (0.77-2.05) | 1.25 (0.77-2.05) |
|  | IgA nephropathy | 12 (3.67) | 4907 (2.60) |  | 1.43 (0.81-2.51) | 1.42 (0.81-2.51) |
| **Natural cause of death** | **Any AID** | 290 (2.56) | 3409 (1.91) |  | 1.27 (1.11-1.44) | 1.27 (1.11-1.44) |
|  | **Endocrine autoimmune diseases** | 144 (2.89) | 3555 (1.93) |  | 1.41 (1.17-1.70) | 1.41 (1.17-1.70) |
|  | Type 1 diabetes | 58 (4.51) | 3641 (1.93) |  | 2.61 (1.71-3.97) | 2.61 (1.71-3.97) |
|  | Autoimmune thyroiditis | 93 (2.41) | 3606 (1.94) |  | 1.22 (0.98-1.51) | 1.21 (0.98-1.51) |
|  | **Inflammatory arthritis** | 31 (2.89) | 3668 (1.95) |  | 1.35 (0.92-1.97) | 1.35 (0.92-1.97) |
|  | Reactive arthritis | 5 (1.94) | 3694 (1.95) |  | 0.82 (0.31-2.19) | 0.82 (0.31-2.19) |
|  | Rheumatoid arthritis | 22 (3.11) | 3677 (1.95) |  | 1.44 (0.91-2.25) | 1.43 (0.91-2.25) |
|  | **Vasculitis** | 13 (3.49) | 3686 (1.95) |  | 1.77 (1.03-3.05) | 1.77 (1.03-3.05) |
|  | Henoch-Schonlein purpura | 8 (2.82) | 3691 (1.95) |  | 1.49 (0.75-2.99) | 1.49 (0.75-2.99) |
|  | **Connective tissue autoimmune diseases** | 21 (4.93) | 3678 (1.95) |  | 2.03 (1.26-3.27) | 2.03 (1.26-3.27) |
|  | Systemic lupus erythematosus | 11 (5.68) | 3688 (1.95) |  | 2.52 (1.35-4.70) | 2.52 (1.35-4.70) |
|  | Systematic sclerosis | 6 (8.18) | 3693 (1.95) |  | 2.12 (0.68-6.58) | 2.12 (0.68-6.58) |
|  | **Skin autoimmune diseases** | 27 (1.98) | 3672 (1.95) |  | 0.87 (0.57-1.32) | 0.87 (0.57-1.32) |
|  | Psoriasis vulgaris | 25 (2.10) | 3674 (1.95) |  | 0.90 (0.58-1.40) | 0.90 (0.58-1.40) |
|  | **Blood autoimmune diseases** | 5 (2.61) | 3694 (1.95) |  | 1.11 (0.42-2.96) | 1.11 (0.42-2.96) |
|  | **Nervous system autoimmune diseases** | 13 (2.97) | 3686 (1.95) |  | 1.63 (0.94-2.81) | 1.63 (0.94-2.81) |
|  | Multiple sclerosis | 11 (3.56) | 3688 (1.95) |  | 1.98 (1.10-3.58) | 1.98 (1.10-3.58) |
|  | **Gastrointestinal autoimmune diseases** | 53 (1.96) | 3646 (1.95) |  | 1.00 (0.75-1.32) | 1.00 (0.75-1.32) |
|  | Crohn disease | 16 (2.01) | 3683 (1.95) |  | 1.08 (0.66-1.77) | 1.08 (0.66-1.77) |
|  | Ulcerative colitis | 20 (1.88) | 3679 (1.95) |  | 0.98 (0.62-1.54) | 0.98 (0.62-1.54) |
|  | Celiac disease | 20 (1.82) | 3679 (1.95) |  | 0.87 (0.55-1.39) | 0.87 (0.55-1.39) |
|  | **Other autoimmune diseases** | 14 (2.82) | 3685 (1.95) |  | 1.43 (0.84-2.41) | 1.43 (0.84-2.41) |
|  | IgA nephropathy | 11 (3.36) | 3688 (1.95) |  | 1.72 (0.95-3.11) | 1.72 (0.95-3.10) |
| **Unnatural cause of death** | **Any AID** | 40 (0.35) | 615 (0.34) |  | 1.27 (0.90-1.79) | 1.27 (0.89-1.79) |
|  | **Endocrine autoimmune diseases** | 18 (0.36) | 637 (0.34) |  | 1.55 (0.92-2.61) | 1.55 (0.92-2.61) |
|  | Type 1 diabetes | 8 (0.62) | 647 (0.34) |  | 2.38 (0.80-7.05) | 2.38 (0.80-7.04) |
|  | Autoimmune thyroiditis | 11 (0.28) | 644 (0.35) |  | 1.40 (0.77-2.55) | 1.40 (0.77-2.55) |
|  | **Gastrointestinal autoimmune diseases** | 15 (0.55) | 640 (0.34) |  | 1.77 (1.02-3.07) | 1.77 (1.02-3.07) |
|  | Ulcerative colitis | 6 (0.56) | 649 (0.34) |  | 1.77 (0.73-4.26) | 1.77 (0.73-4.26) |
|  | Celiac disease | 9 (0.82) | 646 (0.34) |  | 2.85 (1.42-5.74) | 2.85 (1.42-5.73) |

AID: Autoimmune disease; CI: Confidence interval; HR: Hazard ratio; PY: Person-year.

Only autoimmune diseases with ≥ 5 deaths occurring in the exposed group were included in our analyses.

Models 1 were adjusted for offspring’s calendar year of birth, maternal country of birth, parity, age, education, and marital status at the time of birth, maternal smoking in early pregnancy, maternal cardiovascular diseases and diabetes before childbirth, Models 2 were additionally adjusted for maternal family history of cardiovascular diseases.

Supplementary Table S16 Hazard ratios and 95% confidence intervals for mortality in offspring according to maternal autoimmune diseases in offspring with no maternal hypertensive disorders of pregnancy (n=1,390,273)

| Outcome | Exposure | Events (per 10,000 PYs) | |  | HR (95% CI) | |
| --- | --- | --- | --- | --- | --- | --- |
|  |  | Exposed | Non-exposed |  | Model 1 | Model 2 |
| **All cause of death** | **Any AID** | 376 (3.20) | 5343 (2.64) |  | 1.17 (1.06-1.30) | 1.22 (1.09-1.36) |
|  | **Endocrine autoimmune diseases** | 192 (3.57) | 5527 (2.65) |  | 1.28 (1.11-1.48) | 1.35 (1.15-1.58) |
|  | Type 1 diabetes | 71 (5.82) | 5648 (2.65) |  | 2.19 (1.73-2.77) | 2.31 (1.65-3.23) |
|  | Autoimmune thyroiditis | 131 (3.05) | 5588 (2.66) |  | 1.07 (0.90-1.27) | 1.21 (1.01-1.45) |
|  | **Inflammatory arthritis** | 30 (2.83) | 5689 (2.67) |  | 1.04 (0.73-1.49) | 1.02 (0.70-1.51) |
|  | Reactive arthritis | 5 (1.92) | 5714 (2.67) |  | 0.70 (0.29-1.68) | 0.63 (0.24-1.69) |
|  | Rheumatoid arthritis | 22 (3.17) | 5697 (2.67) |  | 1.17 (0.77-1.78) | 1.14 (0.73-1.80) |
|  | **Vasculitis** | 13 (3.59) | 5706 (2.67) |  | 1.34 (0.78-2.30) | 1.32 (0.75-2.33) |
|  | Henoch-Schonlein purpura | 9 (3.27) | 5710 (2.67) |  | 1.22 (0.63-2.34) | 1.19 (0.59-2.37) |
|  | **Connective tissue autoimmune diseases** | 19 (4.44) | 5700 (2.67) |  | 1.66 (1.06-2.60) | 1.53 (0.93-2.50) |
|  | Systemic lupus erythematosus | 7 (3.65) | 5712 (2.67) |  | 1.35 (0.64-2.84) | 1.46 (0.69-3.06) |
|  | Systematic sclerosis | 5 (7.14) | 5714 (2.67) |  | 2.67 (1.11-6.42) | 1.18 (0.29-4.71) |
|  | **Skin autoimmune diseases** | 37 (2.66) | 5682 (2.67) |  | 0.96 (0.70-1.33) | 0.96 (0.68-1.36) |
|  | Psoriasis vulgaris | 33 (2.74) | 5686 (2.67) |  | 0.99 (0.71-1.40) | 0.97 (0.67-1.41) |
|  | **Blood autoimmune diseases** | 6 (2.98) | 5713 (2.67) |  | 1.10 (0.49-2.44) | 1.01 (0.42-2.42) |
|  | **Nervous system autoimmune diseases** | 15 (3.31) | 5704 (2.67) |  | 1.23 (0.74-2.04) | 1.45 (0.87-2.41) |
|  | Multiple sclerosis | 12 (3.76) | 5707 (2.67) |  | 1.39 (0.79-2.45) | 1.69 (0.96-2.97) |
|  | **Gastrointestinal autoimmune diseases** | 77 (2.87) | 5642 (2.67) |  | 1.06 (0.84-1.32) | 1.10 (0.87-1.39) |
|  | Crohn disease | 22 (2.78) | 5697 (2.67) |  | 1.03 (0.68-1.57) | 1.11 (0.72-1.70) |
|  | Ulcerative colitis | 29 (2.73) | 5690 (2.67) |  | 1.01 (0.70-1.46) | 1.05 (0.72-1.55) |
|  | Celiac disease | 32 (2.98) | 5687 (2.67) |  | 1.08 (0.76-1.53) | 1.08 (0.75-1.57) |
|  | **Other autoimmune diseases** | 14 (2.92) | 5705 (2.67) |  | 1.09 (0.65-1.85) | 1.16 (0.69-1.96) |
|  | Sarcoidosis | 5 (3.59) | 5714 (2.67) |  | 1.34 (0.56-3.21) | 1.47 (0.61-3.52) |
|  | IgA nephropathy | 9 (2.93) | 5710 (2.67) |  | 1.11 (0.57-2.13) | 1.18 (0.61-2.27) |
| **Natural cause of death** | **Any AID** | 294 (2.50) | 4012 (1.98) |  | 1.18 (1.05-1.33) | 1.25 (1.10-1.42) |
|  | **Endocrine autoimmune diseases** | 152 (2.82) | 4154 (1.99) |  | 1.29 (1.10-1.52) | 1.39 (1.16-1.66) |
|  | Type 1 diabetes | 56 (4.59) | 4250 (1.99) |  | 2.28 (1.75-2.97) | 2.79 (1.90-4.11) |
|  | Autoimmune thyroiditis | 105 (2.44) | 4201 (2.0) |  | 1.09 (0.90-1.32) | 1.22 (1.00-1.49) |
|  | **Inflammatory arthritis** | 24 (2.26) | 4282 (2.01) |  | 1.08 (0.72-1.61) | 1.08 (0.70-1.65) |
|  | Reactive arthritis | 5 (1.92) | 4301 (2.01) |  | 0.90 (0.37-2.16) | 0.82 (0.31-2.18) |
|  | Rheumatoid arthritis | 16 (2.30) | 4290 (2.01) |  | 1.11 (0.68-1.81) | 1.10 (0.65-1.86) |
|  | **Vasculitis** | 11 (3.04) | 4295 (2.01) |  | 1.48 (0.82-2.68) | 1.61 (0.89-2.91) |
|  | Henoch-Schonlein purpura | 7 (2.54) | 4299 (2.01) |  | 1.24 (0.59-2.61) | 1.39 (0.66-2.91) |
|  | **Connective tissue autoimmune diseases** | 17 (3.97) | 4289 (2.01) |  | 1.95 (1.21-3.14) | 1.73 (1.02-2.93) |
|  | Systemic lupus erythematosus | 7 (3.65) | 4299 (2.01) |  | 1.77 (0.85-3.73) | 1.87 (0.89-3.93) |
|  | Systematic sclerosis | 5 (7.14) | 4301 (2.01) |  | 3.54 (1.47-8.52) | 1.56 (0.39-6.24) |
|  | **Skin autoimmune diseases** | 29 (2.08) | 4277 (2.01) |  | 0.96 (0.67-1.39) | 0.94 (0.63-1.40) |
|  | Psoriasis vulgaris | 26 (2.16) | 4280 (2.01) |  | 1.00 (0.68-1.47) | 0.94 (0.61-1.45) |
|  | **Blood autoimmune diseases** | 5 (2.48) | 4301 (2.01) |  | 1.19 (0.49-2.86) | 1.05 (0.39-2.80) |
|  | **Nervous system autoimmune diseases** | 13 (2.87) | 4293 (2.01) |  | 1.39 (0.80-2.39) | 1.61 (0.93-2.78) |
|  | Multiple sclerosis | 11 (3.45) | 4295 (2.01) |  | 1.65 (0.92-2.99) | 1.96 (1.08-3.54) |
|  | **Gastrointestinal autoimmune diseases** | 57 (2.12) | 4249 (2.01) |  | 1.01 (0.78-1.32) | 1.11 (0.85-1.46) |
|  | Crohn disease | 19 (2.40) | 4287 (2.01) |  | 1.17 (0.75-1.84) | 1.32 (0.84-2.06) |
|  | Ulcerative colitis | 21 (1.98) | 4285 (2.01) |  | 0.95 (0.62-1.47) | 1.05 (0.68-1.63) |
|  | Celiac disease | 21 (1.95) | 4285 (2.01) |  | 0.91 (0.59-1.40) | 0.97 (0.62-1.53) |
|  | **Other autoimmune diseases** | 12 (2.50) | 4294 (2.01) |  | 1.24 (0.70-2.19) | 1.30 (0.74-2.29) |
|  | IgA nephropathy | 8 (2.61) | 4298 (2.01) |  | 1.31 (0.65-2.62) | 1.39 (0.69-2.78) |
| **Unnatural cause of death** | **Any AID** | 43 (0.36) | 725 (0.36) |  | 1.24 (0.91-1.69) | 1.20 (0.86-1.69) |
|  | **Endocrine autoimmune diseases** | 19 (0.35) | 749 (0.36) |  | 1.27 (0.80-2.00) | 1.30 (0.79-2.14) |
|  | Type 1 diabetes | 9 (0.73) | 759 (0.36) |  | 2.16 (1.12-4.17) | 1.74 (0.72-4.22) |
|  | Autoimmune thyroiditis | 11 (0.25) | 757 (0.36) |  | 0.98 (0.54-1.78) | 1.17 (0.64-2.14) |
|  | **Gastrointestinal autoimmune diseases** | 15 (0.56) | 753 (0.35) |  | 1.80 (1.08-3.00) | 1.73 (1.00-3.01) |
|  | Ulcerative colitis | 6 (0.56) | 762 (0.36) |  | 1.78 (0.80-3.98) | 1.72 (0.71-4.15) |
|  | Celiac disease | 9 (0.84) | 759 (0.35) |  | 2.92 (1.51-5.63) | 2.78 (1.38-5.59) |

AID: Autoimmune disease; CI: Confidence interval; HR: Hazard ratio; PY: Person-year.

Only autoimmune diseases with ≥ 5 deaths occurring in the exposed group were included in our analyses.

Models 1 were adjusted for age (as the underlying time scale in the Cox regression models); Models 2 were additionally adjusted for offspring’s calendar year of birth, parity, maternal age, country of birth, education, and marital status at the time of birth, maternal smoking in early pregnancy, maternal cardiovascular diseases and diabetes before childbirth.

Supplementary Table S17 Hazard ratios and 95% confidence intervals for mortality in offspring according to maternal autoimmune diseases in offspring without autoimmune diseases (n= 1,392,795)

| Outcome | Exposure | Events (per 10,000 PYs) | |  | HR (95% CI) | |
| --- | --- | --- | --- | --- | --- | --- |
|  |  | Exposed | Non-exposed |  | Model 1 | Model 2 |
| **All cause of death** | **Any AID** | 412 (3.49) | 5522 (2.74) |  | 1.32 (1.19-1.47) | 1.25 (1.12-1.39) |
|  | **Endocrine autoimmune diseases** | 214 (3.84) | 5720 (2.75) |  | 1.47 (1.27-1.70) | 1.35 (1.16-1.57) |
|  | Type 1 diabetes | 84 (6.12) | 5850 (2.76) |  | 2.27 (1.82-2.83) | 2.12 (1.56-2.90) |
|  | Autoimmune thyroiditis | 142 (3.26) | 5792 (2.77) |  | 1.23 (1.03-1.47) | 1.20 (1.01-1.44) |
|  | **Inflammatory arthritis** | 39 (3.70) | 5895 (2.77) |  | 1.28 (0.91-1.81) | 1.25 (0.89-1.77) |
|  | Reactive arthritis | 5 (1.91) | 5929 (2.78) |  | 0.62 (0.23-1.65) | 0.61 (0.23-1.62) |
|  | Rheumatoid arthritis | 30 (4.36) | 5904 (2.77) |  | 1.50 (1.01-2.22) | 1.46 (0.98-2.16) |
|  | **Vasculitis** | 15 (4.10) | 5919 (2.78) |  | 1.51 (0.90-2.56) | 1.44 (0.85-2.43) |
|  | Henoch-Schonlein purpura | 10 (3.63) | 5924 (2.78) |  | 1.30 (0.67-2.50) | 1.27 (0.66-2.44) |
|  | **Connective tissue autoimmune diseases** | 25 (5.82) | 5909 (2.77) |  | 2.01 (1.31-3.09) | 1.88 (1.22-2.89) |
|  | Systemic lupus erythematosus | 11 (5.73) | 5923 (2.78) |  | 2.17 (1.16-4.03) | 1.94 (1.04-3.61) |
|  | Systematic sclerosis | 6 (8.44) | 5928 (2.78) |  | 1.73 (0.56-5.38) | 1.63 (0.52-5.05) |
|  | **Skin autoimmune diseases** | 38 (2.76) | 5896 (2.78) |  | 0.97 (0.69-1.37) | 0.96 (0.68-1.36) |
|  | Psoriasis vulgaris | 35 (2.93) | 5899 (2.78) |  | 1.02 (0.71-1.46) | 1.01 (0.70-1.44) |
|  | **Blood autoimmune diseases** | 7 (3.50) | 5927 (2.78) |  | 1.00 (0.42-2.41) | 0.97 (0.40-2.33) |
|  | **Nervous system autoimmune diseases** | 14 (3.11) | 5920 (2.78) |  | 1.32 (0.78-2.23) | 1.30 (0.77-2.19) |
|  | Multiple sclerosis | 11 (3.47) | 5923 (2.78) |  | 1.50 (0.83-2.71) | 1.48 (0.82-2.68) |
|  | **Gastrointestinal autoimmune diseases** | 76 (2.96) | 5858 (2.78) |  | 1.10 (0.87-1.40) | 1.08 (0.85-1.37) |
|  | Crohn disease | 21 (2.71) | 5913 (2.78) |  | 1.05 (0.68-1.63) | 1.04 (0.67-1.61) |
|  | Ulcerative colitis | 29 (2.78) | 5905 (2.78) |  | 1.04 (0.71-1.53) | 1.03 (0.70-1.51) |
|  | Celiac disease | 32 (3.24) | 5902 (2.78) |  | 1.15 (0.79-1.67) | 1.13 (0.78-1.63) |
|  | **Other autoimmune diseases** | 18 (3.52) | 5916 (2.78) |  | 1.40 (0.88-2.22) | 1.31 (0.83-2.09) |
|  | Sarcoidosis | 5 (3.60) | 5929 (2.78) |  | 1.47 (0.61-3.53) | 1.41 (0.59-3.39) |
|  | IgA nephropathy | 12 (3.55) | 5922 (2.78) |  | 1.40 (0.79-2.46) | 1.33 (0.76-2.35) |
| **Natural cause of death** | **Any AID** | 330 (2.79) | 4173 (2.07) |  | 1.39 (1.23-1.56) | 1.32 (1.17-1.49) |
|  | **Endocrine autoimmune diseases** | 173 (3.10) | 4330 (2.08) |  | 1.53 (1.30-1.79) | 1.42 (1.19-1.68) |
|  | Type 1 diabetes | 68 (4.95) | 4435 (2.09) |  | 2.47 (1.93-3.15) | 2.54 (1.78-3.62) |
|  | Autoimmune thyroiditis | 115 (2.64) | 4388 (2.10) |  | 1.27 (1.04-1.54) | 1.24 (1.02-1.51) |
|  | **Inflammatory arthritis** | 31 (2.94) | 4472 (2.10) |  | 1.36 (0.93-1.99) | 1.33 (0.91-1.94) |
|  | Reactive arthritis | 5 (1.91) | 4498 (2.11) |  | 0.80 (0.30-2.13) | 0.78 (0.29-2.08) |
|  | Rheumatoid arthritis | 22 (3.20) | 4481 (2.10) |  | 1.48 (0.94-2.32) | 1.43 (0.91-2.25) |
|  | **Vasculitis** | 13 (3.55) | 4490 (2.10) |  | 1.87 (1.08-3.22) | 1.76 (1.02-3.04) |
|  | Henoch-Schonlein purpura | 8 (2.91) | 4495 (2.11) |  | 1.53 (0.77-3.07) | 1.50 (0.75-2.99) |
|  | **Connective tissue autoimmune diseases** | 23 (5.35) | 4480 (2.10) |  | 2.36 (1.50-3.70) | 2.19 (1.39-3.44) |
|  | Systemic lupus erythematosus | 11 (5.73) | 4492 (2.10) |  | 2.79 (1.50-5.20) | 2.47 (1.33-4.60) |
|  | Systematic sclerosis | 6 (8.44) | 4497 (2.10) |  | 2.29 (0.74-7.11) | 2.13 (0.69-6.61) |
|  | **Skin autoimmune diseases** | 30 (2.17) | 4473 (2.11) |  | 0.95 (0.64-1.41) | 0.94 (0.63-1.39) |
|  | Psoriasis vulgaris | 27 (2.26) | 4476 (2.11) |  | 0.96 (0.63-1.47) | 0.95 (0.63-1.45) |
|  | **Blood autoimmune diseases** | 6 (2.99) | 4497 (2.11) |  | 1.05 (0.39-2.80) | 1.01 (0.38-2.68) |
|  | **Nervous system autoimmune diseases** | 13 (2.89) | 4490 (2.10) |  | 1.56 (0.91-2.69) | 1.53 (0.89-2.64) |
|  | Multiple sclerosis | 11 (3.47) | 4492 (2.10) |  | 1.89 (1.05-3.41) | 1.87 (1.03-3.38) |
|  | **Gastrointestinal autoimmune diseases** | 59 (2.29) | 4444 (2.10) |  | 1.16 (0.89-1.52) | 1.15 (0.88-1.49) |
|  | Crohn disease | 19 (2.45) | 4484 (2.11) |  | 1.30 (0.83-2.05) | 1.28 (0.82-2.01) |
|  | Ulcerative colitis | 22 (2.10) | 4481 (2.11) |  | 1.08 (0.71-1.66) | 1.07 (0.70-1.64) |
|  | Celiac disease | 22 (2.22) | 4481 (2.11) |  | 1.08 (0.70-1.68) | 1.06 (0.68-1.64) |
|  | **Other autoimmune diseases** | 15 (2.93) | 4488 (2.10) |  | 1.52 (0.92-2.53) | 1.42 (0.85-2.36) |
|  | IgA nephropathy | 11 (3.25) | 4492 (2.10) |  | 1.69 (0.94-3.06) | 1.60 (0.89-2.90) |
| **Unnatural cause of death** | **Any AID** | 38 (0.32) | 721 (0.36) |  | 1.12 (0.79-1.59) | 1.04 (0.72-1.49) |
|  | **Endocrine autoimmune diseases** | 18 (0.32) | 741 (0.35) |  | 1.38 (0.86-2.20) | 1.19 (0.71-2.00) |
|  | Type 1 diabetes | 9 (0.65) | 750 (0.35) |  | 2.05 (1.06-3.95) | 1.63 (0.67-3.96) |
|  | Autoimmune thyroiditis | 10 (0.23) | 749 (0.36) |  | 1.10 (0.59-2.06) | 1.06 (0.57-2.00) |
|  | **Gastrointestinal autoimmune diseases** | 13 (0.50) | 746 (0.35) |  | 1.57 (0.87-2.85) | 1.56 (0.86-2.83) |
|  | Ulcerative colitis | 5 (0.48) | 754 (0.35) |  | 1.42 (0.53-3.80) | 1.42 (0.53-3.79) |
|  | Celiac disease | 9 (0.91) | 750 (0.35) |  | 3.16 (1.57-6.36) | 3.12 (1.55-6.27) |

AID: Autoimmune disease; CI: Confidence interval; HR: Hazard ratio; PY: Person-year.

Only autoimmune diseases with ≥ 5 deaths occurring in the exposed group were included in our analyses.

Models 1 were adjusted for age (as the underlying time scale in the Cox regression models); Models 2 were additionally adjusted for offspring’s calendar year of birth, parity, maternal age, country of birth, education, and marital status at the time of birth, maternal smoking in early pregnancy, maternal cardiovascular diseases and diabetes before childbirth.

Supplementary Table S18 Hazard ratios and 95% confidence intervals for mortality in offspring according to maternal autoimmune diseases defined as having at least two diagnosis records for each specific autoimmune disease

| Outcome | Exposure | Events (per 10,000 PYs) | | HR (95% CI) |
| --- | --- | --- | --- | --- |
|  |  | Exposed | Non-exposed |  |
| **All cause of death** | **Any AID** | 312 (3.54) | 5842 (2.71) | 1.29 (1.14-1.46) |
|  | **Endocrine autoimmune diseases** | 176 (3.91) | 5978 (2.72) | 1.40 (1.19-1.66) |
|  | Type 1 diabetes | 70 (5.82) | 6084 (2.73) | 2.02 (1.47-2.80) |
|  | Autoimmune thyroiditis | 113 (3.29) | 6041 (2.74) | 1.22 (1.00-1.49) |
|  | **Inflammatory arthritis** | 35 (3.81) | 6119 (2.74) | 1.33 (0.93-1.91) |
|  | Rheumatoid arthritis | 30 (4.18) | 6124 (2.74) | 1.42 (0.96-2.10) |
|  | **Connective tissue autoimmune diseases** | 19 (5.09) | 6135 (2.74) | 1.75 (1.09-2.82) |
|  | Systemic lupus erythematosus | 11 (5.79) | 6143 (2.74) | 1.99 (1.07-3.69) |
|  | **Skin autoimmune diseases** | 35 (2.87) | 6119 (2.74) | 1.00 (0.70-1.43) |
|  | Psoriasis vulgaris | 34 (2.82) | 6120 (2.74) | 0.97 (0.68-1.40) |
|  | **Nervous system autoimmune diseases** | 10 (3.21) | 6144 (2.74) | 1.34 (0.72-2.50) |
|  | Multiple sclerosis | 9 (3.56) | 6145 (2.74) | 1.52 (0.79-2.93) |
|  | **Gastrointestinal autoimmune diseases** | 45 (3.07) | 6109 (2.74) | 1.14 (0.83-1.54) |
|  | Crohn disease | 16 (2.98) | 6138 (2.74) | 1.14 (0.69-1.89) |
|  | Ulcerative colitis | 15 (2.18) | 6139 (2.75) | 0.84 (0.50-1.43) |
|  | Celiac disease | 16 (4.98) | 6138 (2.74) | 1.65 (0.98-2.79) |
|  | **Other autoimmune diseases** | 8 (3.09) | 6146 (2.74) | 1.13 (0.57-2.27) |
|  | IgA nephropathy | 6 (3.46) | 6148 (2.74) | 1.28 (0.57-2.85) |
| **Natural cause of death** | **Any AID** | 248 (2.81) | 4419 (2.05) | 1.34 (1.17-1.54) |
|  | **Endocrine autoimmune diseases** | 147 (3.27) | 4520 (2.06) | 1.52 (1.27-1.83) |
|  | Type 1 diabetes | 57 (4.74) | 4610 (2.07) | 2.44 (1.69-3.51) |
|  | Autoimmune thyroiditis | 95 (2.77) | 4572 (2.07) | 1.30 (1.05-1.61) |
|  | **Inflammatory arthritis** | 27 (2.94) | 4640 (2.08) | 1.38 (0.92-2.06) |
|  | Rheumatoid arthritis | 22 (3.06) | 4645 (2.08) | 1.40 (0.89-2.19) |
|  | **Connective tissue autoimmune diseases** | 17 (4.55) | 4650 (2.08) | 2.00 (1.20-3.32) |
|  | Systemic lupus erythematosus | 11 (5.79) | 4656 (2.08) | 2.54 (1.36-4.73) |
|  | **Skin autoimmune diseases** | 28 (2.30) | 4639 (2.08) | 0.98 (0.65-1.48) |
|  | Psoriasis vulgaris | 27 (2.24) | 4640 (2.08) | 0.95 (0.63-1.45) |
|  | **Nervous system autoimmune diseases** | 9 (2.89) | 4658 (2.08) | 1.53 (0.79-2.94) |
|  | Multiple sclerosis | 8 (3.16) | 4659 (2.08) | 1.70 (0.85-3.41) |
|  | **Gastrointestinal autoimmune diseases** | 32 (2.18) | 4635 (2.08) | 1.12 (0.79-1.59) |
|  | Crohn disease | 13 (2.42) | 4654 (2.08) | 1.29 (0.75-2.22) |
|  | Ulcerative colitis | 10 (1.45) | 4657 (2.08) | 0.78 (0.42-1.44) |
|  | Celiac disease | 10 (3.11) | 4657 (2.08) | 1.41 (0.73-2.71) |
|  | **Other autoimmune diseases** | 5 (1.93) | 4662 (2.08) | 0.92 (0.38-2.22) |
|  | IgA nephropathy | 5 (2.88) | 4662 (2.08) | 1.40 (0.58-3.38) |
| **Unnatural cause of death** | **Any AID** | 25 (0.28) | 769 (0.36) | 0.94 (0.61-1.45) |
|  | **Endocrine autoimmune diseases** | 11 (0.24) | 783 (0.36) | 0.88 (0.46-1.68) |
|  | Type 1 diabetes | 5 (0.41) | 789 (0.35) | 0.83 (0.30-2.31) |
|  | Autoimmune thyroiditis | 7 (0.20) | 787 (0.36) | 1.00 (0.48-2.12) |
|  | **Gastrointestinal autoimmune diseases** | 8 (0.54) | 786 (0.35) | 1.68 (0.80-3.54) |

AID: Autoimmune disease; CI: Confidence interval; HR: Hazard ratio; PY: Person-year.

Only autoimmune diseases with ≥ 5 deaths occurring in the exposed group were included in our analyses.

Analyses were adjusted for offspring’s calendar year of birth, parity, maternal country of birth, age, education, and marital status at the time of birth, maternal smoking in early pregnancy, maternal cardiovascular diseases and diabetes before childbirth.

Supplementary Table S19 Hazard ratios and 95% confidence intervals for mortality in offspring according to maternal exposure to only one autoimmune disease (n=1,446,733)

| Outcome | Exposure | Events (per 10,000 PYs) | | HR (95% CI) |
| --- | --- | --- | --- | --- |
|  |  | Exposed | Non-exposed |  |
| **All cause of death** | **Any AID** | 376 (3.27) | 5728 (2.71) | 1.21 (1.08-1.35) |
|  | **Endocrine autoimmune diseases** | 192 (3.57) | 5912 (2.72) | 1.29 (1.10-1.52) |
|  | Type 1 diabetes | 67 (5.50) | 6037 (2.72) | 1.89 (1.36-2.63) |
|  | Autoimmune thyroiditis | 125 (3.02) | 5979 (2.73) | 1.16 (0.96-1.39) |
|  | **Inflammatory arthritis** | 26 (3.0) | 6078 (2.74) | 1.05 (0.69-1.59) |
|  | Reactive arthritis | 5 (2.36) | 6099 (2.74) | 0.77 (0.29-2.04) |
|  | Rheumatoid arthritis | 18 (3.33) | 6086 (2.74) | 1.14 (0.69-1.90) |
|  | **Vasculitis** | 14 (4.31) | 6090 (2.74) | 1.57 (0.91-2.70) |
|  | Henoch-Schonlein purpura | 10 (3.92) | 6094 (2.74) | 1.42 (0.74-2.72) |
|  | **Connective tissue autoimmune diseases** | 16 (5.28) | 6088 (2.73) | 1.84 (1.09-3.11) |
|  | Systemic lupus erythematosus | 5 (4.53) | 6099 (2.74) | 1.75 (0.73-4.21) |
|  | Systematic sclerosis | 5 (7.98) | 6099 (2.74) | 1.95 (0.63-6.06) |
|  | **Skin autoimmune diseases** | 31 (2.43) | 6073 (2.74) | 0.89 (0.62-1.29) |
|  | Psoriasis vulgaris | 28 (2.50) | 6076 (2.74) | 0.91 (0.61-1.35) |
|  | **Nervous system autoimmune diseases** | 14 (3.45) | 6090 (2.74) | 1.47 (0.87-2.48) |
|  | Multiple sclerosis | 11 (3.88) | 6093 (2.74) | 1.69 (0.94-3.05) |
|  | **Gastrointestinal autoimmune diseases** | 67 (2.91) | 6037 (2.74) | 1.09 (0.85-1.41) |
|  | Crohn disease | 15 (2.76) | 6089 (2.74) | 1.12 (0.67-1.85) |
|  | Ulcerative colitis | 24 (2.97) | 6080 (2.74) | 1.15 (0.76-1.75) |
|  | Celiac disease | 28 (2.95) | 6076 (2.74) | 1.03 (0.69-1.54) |
|  | **Other autoimmune diseases** | 14 (3.12) | 6090 (2.74) | 1.19 (0.71-2.02) |
|  | Sarcoidosis | 5 (3.97) | 6099 (2.74) | 1.57 (0.65-3.78) |
|  | IgA nephropathy | 8 (2.69) | 6096 (2.74) | 1.04 (0.52-2.09) |
| **Natural cause of death** | **Any AID** | 291 (2.53) | 4331 (2.05) | 1.22 (1.07-1.39) |
|  | **Endocrine autoimmune diseases** | 151 (2.80) | 4471 (2.05) | 1.31 (1.10-1.57) |
|  | Type 1 diabetes | 53 (4.34) | 4569 (2.06) | 2.16 (1.48-3.15) |
|  | Autoimmune thyroiditis | 98 (2.36) | 4524 (2.07) | 1.14 (0.93-1.41) |
|  | **Inflammatory arthritis** | 18 (2.07) | 4604 (2.07) | 0.99 (0.61-1.62) |
|  | Reactive arthritis | 5 (2.36) | 4617 (2.07) | 0.99 (0.37-2.64) |
|  | Rheumatoid arthritis | 10 (1.84) | 4612 (2.07) | 0.90 (0.47-1.73) |
|  | **Vasculitis** | 12 (3.69) | 4610 (2.07) | 1.92 (1.09-3.38) |
|  | Henoch-Schonlein purpura | 8 (3.13) | 4614 (2.07) | 1.68 (0.84-3.35) |
|  | **Connective tissue autoimmune diseases** | 14 (4.62) | 4608 (2.07) | 2.05 (1.16-3.62) |
|  | Systemic lupus erythematosus | 5 (4.53) | 4617 (2.07) | 2.26 (0.94-5.42) |
|  | Systematic sclerosis | 5 (7.98) | 4617 (2.07) | 2.59 (0.84-8.04) |
|  | **Skin autoimmune diseases** | 23 (1.80) | 4599 (2.07) | 0.82 (0.53-1.28) |
|  | Psoriasis vulgaris | 21 (1.88) | 4601 (2.07) | 0.85 (0.53-1.35) |
|  | **Nervous system autoimmune diseases** | 12 (2.95) | 4610 (2.07) | 1.61 (0.91-2.84) |
|  | Multiple sclerosis | 10 (3.52) | 4612 (2.07) | 1.95 (1.05-3.62) |
|  | **Gastrointestinal autoimmune diseases** | 49 (2.13) | 4573 (2.07) | 1.08 (0.81-1.44) |
|  | Crohn disease | 13 (2.40) | 4609 (2.07) | 1.27 (0.74-2.20) |
|  | Ulcerative colitis | 18 (2.22) | 4604 (2.07) | 1.15 (0.72-1.85) |
|  | Celiac disease | 18 (1.89) | 4604 (2.07) | 0.91 (0.55-1.48) |
|  | **Other autoimmune diseases** | 11 (2.45) | 4611 (2.07) | 1.22 (0.68-2.21) |
|  | IgA nephropathy | 7 (2.35) | 4615 (2.07) | 1.20 (0.57-2.53) |
| **Unnatural cause of death** | **Any AID** | 42 (0.36) | 750 (0.35) | 1.22 (0.87-1.71) |
|  | **Endocrine autoimmune diseases** | 19 (0.35) | 773 (0.35) | 1.35 (0.82-2.22) |
|  | Type 1 diabetes | 8 (0.65) | 784 (0.35) | 1.61 (0.64-4.04) |
|  | Autoimmune thyroiditis | 11 (0.26) | 781 (0.36) | 1.26 (0.69-2.29) |
|  | **Gastrointestinal autoimmune diseases** | 14 (0.61) | 778 (0.35) | 1.85 (1.05-3.28) |
|  | Ulcerative colitis | 5 (0.62) | 787 (0.35) | 1.76 (0.66-4.71) |
|  | Celiac disease | 8 (0.84) | 784 (0.35) | 2.79 (1.32-5.89) |

AID: Autoimmune disease; CI: Confidence interval; HR: Hazard ratio; PY: Person-year.

Only autoimmune diseases with ≥ 5 deaths occurring in the exposed group were included in our analyses.

Analyses were adjusted for offspring’s calendar year of birth, parity, maternal country of birth, age, education, and marital status at the time of birth, maternal smoking in early pregnancy, maternal cardiovascular diseases and diabetes before childbirth.

Supplementary Table S20 Hazard ratios and 95% confidence intervals for all-cause mortality in offspring according to maternal autoimmune diseases during pregnancy accounting for within-family correlation among births by the same mother

| Exposure | Events (per 10,000 PYs) | |  | HR (95% CI) | | |
| --- | --- | --- | --- | --- | --- | --- |
|  | Exposed | Non-exposed |  | Model 1 | Model 2 | Model 3 |
| **Any autoimmune diseases** | 426 (3.35) | 5728 (2.71) |  | 1.19 (1.08-1.32) | 1.28 (1.15-1.43) | 1.22 (1.09-1.36) |
| **Endocrine autoimmune diseases** | 220 (3.68) | 5934 (2.72) |  | 1.28 (1.12-1.47) | 1.42 (1.23-1.64) | 1.31 (1.12-1.53) |
| Type 1 diabetes | 84 (5.59) | 6070 (2.73) |  | 2.04 (1.64-2.54) | 2.10 (1.68-2.64) | 1.77 (1.32-2.37) |
| Autoimmune thyroiditis | 148 (3.18) | 6006 (2.74) |  | 1.09 (0.92-1.29) | 1.22 (1.02-1.46) | 1.19 (1.00-1.43) |
| **Inflammatory arthritis** | 40 (3.52) | 6114 (2.74) |  | 1.26 (0.92-1.73) | 1.24 (0.88-1.76) | 1.21 (0.86-1.71) |
| Reactive arthritis | 5 (1.81) | 6149 (2.75) |  | 0.64 (0.27-1.54) | 0.59 (0.22-1.58) | 0.58 (0.22-1.52) |
| Rheumatoid arthritis | 31 (4.15) | 6123 (2.74) |  | 1.49 (1.04-2.14) | 1.45 (0.98-2.16) | 1.41 (0.95-2.10) |
| **Vasculitis autoimmune diseases** | 15 (3.87) | 6139 (2.74) |  | 1.40 (0.77-2.54) | 1.45 (0.78-2.71) | 1.37 (0.73-2.56) |
| Henoch-Schonlein purpura | 10 (3.42) | 6144 (2.74) |  | 1.24 (0.60-2.57) | 1.24 (0.57-2.72) | 1.20 (0.55-2.64) |
| **Connective tissue autoimmune diseases** | 25 (5.36) | 6129 (2.74) |  | 1.95 (1.26-3.01) | 1.87 (1.18-2.98) | 1.76 (1.10-2.80) |
| Systemic lupus erythematosus | 11 (5.16) | 6143 (2.74) |  | 1.86 (0.93-3.71) | 1.98 (0.95-4.12) | 1.78 (0.85-3.71) |
| Systematic sclerosis | 6 (7.90) | 6148 (2.74) |  | 2.88 (1.15-7.23) | 1.64 (0.54-5.05) | 1.57 (0.51-4.82) |
| **Skin autoimmune diseases** | 39 (2.64) | 6115 (2.75) |  | 0.93 (0.67-1.28) | 0.94 (0.67-1.34) | 0.93 (0.66-1.32) |
| Psoriasis vulgaris | 35 (2.73) | 6119 (2.75) |  | 0.96 (0.69-1.36) | 0.96 (0.66-1.39) | 0.95 (0.65-1.37) |
| **Blood autoimmune diseases** | 7 (3.26) | 6147 (2.74) |  | 1.17 (0.56-2.44) | 0.95 (0.40-2.27) | 0.92 (0.38-2.21) |
| **Nervous autoimmune diseases** | 16 (3.38) | 6138 (2.74) |  | 1.22 (0.72-2.04) | 1.45 (0.86-2.43) | 1.42 (0.85-2.39) |
| Multiple sclerosis | 13 (3.90) | 6141 (2.74) |  | 1.40 (0.78-2.51) | 1.70 (0.95-3.05) | 1.69 (0.94-3.01) |
| **Gastrointestinal autoimmune diseases** | 80 (2.83) | 6074 (2.74) |  | 1.01 (0.81-1.26) | 1.07 (0.85-1.35) | 1.06 (0.84-1.33) |
| Crohn disease | 23 (2.75) | 6131 (2.75) |  | 0.99 (0.66-1.50) | 1.08 (0.71-1.64) | 1.06 (0.70-1.62) |
| Ulcerative colitis | 30 (2.68) | 6124 (2.75) |  | 0.96 (0.67-1.39) | 1.02 (0.69-1.51) | 1.01 (0.68-1.49) |
| Celiac disease | 33 (2.93) | 6121 (2.74) |  | 1.03 (0.73-1.45) | 1.06 (0.73-1.52) | 1.04 (0.72-1.49) |
| **Other autoimmune diseases** | 18 (3.32) | 6136 (2.74) |  | 1.21 (0.76-1.92) | 1.34 (0.84-2.12) | 1.26 (0.80-2.00) |
| Sarcoidosis | 5 (3.36) | 6149 (2.74) |  | 1.22 (0.51-2.92) | 1.39 (0.58-3.33) | 1.33 (0.56-3.20) |
| IgA nephropathy | 12 (3.37) | 6142 (2.74) |  | 1.23 (0.70-2.17) | 1.34 (0.76-2.36) | 1.29 (0.73-2.26) |

CI: Confidence interval; HR: Hazard ratio; PY: Person-year.

Only autoimmune diseases with ≥ 5 deaths occurring in the exposed group were included in our analyses.

Models 1 were adjusted for offspring age (as the underlying time scale in the Cox regression models); Models 2 were additionally adjusted for offspring’s calendar year of birth, parity, maternal age, country of birth, education, and marital status at the time of birth; Models 3 were adjusted for factors in model 2 and for maternal smoking in early pregnancy, maternal cardiovascular diseases and type 2 diabetes before childbirth.

Supplementary Table S21 Hazard ratios and 95% confidence intervals for mortality in offspring according to maternal autoimmune diseases diagnosed within three years postpartum

| Outcome | Exposure | Events (per 10,000 PYs) | | HR (95% CI) |
| --- | --- | --- | --- | --- |
|  |  | Exposed | Non-exposed |  |
| **All cause of death** | **Any AID** | 584 (3.51) | 5570 (2.68) | 1.29 (1.18-1.42) |
|  | **Endocrine autoimmune diseases** | 324 (4.17) | 5830 (2.69) | 1.53 (1.35-1.74) |
|  | Type 1 diabetes | 99 (5.81) | 6055 (2.72) | 2.13 (1.59-2.86) |
|  | Autoimmune thyroiditis | 246 (3.89) | 5908 (2.71) | 1.47 (1.28-1.68) |
|  | **Inflammatory arthritis** | 49 (3.14) | 6105 (2.74) | 1.11 (0.82-1.49) |
|  | Reactive arthritis | 9 (2.32) | 6145 (2.75) | 0.83 (0.41-1.65) |
|  | Rheumatoid arthritis | 35 (3.46) | 6119 (2.74) | 1.18 (0.83-1.70) |
|  | Ankylosing spondylitis | 5 (2.15) | 6149 (2.75) | 0.87 (0.36-2.09) |
|  | **Vasculitis** | 16 (3.8) | 6138 (2.74) | 1.28 (0.76-2.16) |
|  | Henoch-Schonlein purpura | 11 (3.63) | 6143 (2.74) | 1.17 (0.61-2.26) |
|  | **Connective tissue autoimmune diseases** | 33 (5.64) | 6121 (2.74) | 1.87 (1.29-2.71) |
|  | Systemic lupus erythematosus | 17 (6.39) | 6137 (2.74) | 2.27 (1.39-3.72) |
|  | Systematic sclerosis | 7 (8.34) | 6147 (2.74) | 1.42 (0.46-4.40) |
|  | Sjögren’s disease | 5 (3.41) | 6149 (2.74) | 1.42 (0.59-3.40) |
|  | **Skin autoimmune diseases** | 66 (2.75) | 6088 (2.75) | 1.00 (0.77-1.29) |
|  | Psoriasis vulgaris | 61 (2.90) | 6093 (2.74) | 1.04 (0.80-1.36) |
|  | **Blood autoimmune diseases** | 13 (4.75) | 6141 (2.74) | 1.43 (0.77-2.66) |
|  | Idiopathic thrombocytopenic purpura | 9 (4.16) | 6145 (2.74) | 1.27 (0.61-2.67) |
|  | **Nervous system autoimmune diseases** | 23 (3.37) | 6131 (2.74) | 1.29 (0.84-1.98) |
|  | Multiple sclerosis | 18 (3.66) | 6136 (2.74) | 1.47 (0.91-2.37) |
|  | **Gastrointestinal autoimmune diseases** | 92 (2.69) | 6062 (2.75) | 0.98 (0.79-1.22) |
|  | Crohn disease | 27 (2.64) | 6127 (2.75) | 0.98 (0.66-1.45) |
|  | Ulcerative colitis | 34 (2.38) | 6120 (2.75) | 0.90 (0.63-1.27) |
|  | Celiac disease | 37 (2.83) | 6117 (2.74) | 1.02 (0.72-1.43) |
|  | **Other autoimmune diseases** | 25 (3.45) | 6129 (2.74) | 1.30 (0.88-1.93) |
|  | Sarcoidosis | 9 (3.57) | 6145 (2.74) | 1.39 (0.72-2.67) |
|  | IgA nephropathy | 15 (3.47) | 6139 (2.74) | 1.32 (0.79-2.19) |
| **Natural cause of death** | **Any AID** | 462 (2.77) | 4205 (2.02) | 1.34 (1.20-1.48) |
|  | **Endocrine autoimmune diseases** | 261 (3.35) | 4406 (2.03) | 1.59 (1.38-1.82) |
|  | Type 1 diabetes | 80 (4.69) | 4587 (2.06) | 2.47 (1.77-3.44) |
|  | Autoimmune thyroiditis | 200 (3.16) | 4467 (2.05) | 1.52 (1.31-1.76) |
|  | **Inflammatory arthritis** | 41 (2.63) | 4626 (2.08) | 1.24 (0.90-1.71) |
|  | Reactive arthritis | 9 (2.32) | 4658 (2.08) | 1.07 (0.54-2.14) |
|  | Rheumatoid arthritis | 27 (2.67) | 4640 (2.08) | 1.24 (0.83-1.85) |
|  | Ankylosing spondylitis | 5 (2.15) | 4662 (2.08) | 1.12 (0.47-2.70) |
|  | **Vasculitis** | 14 (3.32) | 4653 (2.08) | 1.56 (0.91-2.70) |
|  | Henoch-Schonlein purpura | 9 (2.97) | 4658 (2.08) | 1.38 (0.69-2.76) |
|  | **Connective tissue autoimmune diseases** | 31 (5.3) | 4636 (2.07) | 2.24 (1.52-3.30) |
|  | Systemic lupus erythematosus | 17 (6.39) | 4650 (2.08) | 2.92 (1.78-4.77) |
|  | Systematic sclerosis | 7 (8.34) | 4660 (2.08) | 1.86 (0.60-5.76) |
|  | Sjögren’s disease | 5 (3.41) | 4662 (2.08) | 1.81 (0.75-4.35) |
|  | **Skin autoimmune diseases** | 50 (2.08) | 4617 (2.08) | 0.95 (0.71-1.29) |
|  | Psoriasis vulgaris | 46 (2.18) | 4621 (2.08) | 0.99 (0.73-1.35) |
|  | **Blood autoimmune diseases** | 11 (4.02) | 4656 (2.08) | 1.48 (0.74-2.97) |
|  | Idiopathic thrombocytopenic purpura | 7 (3.24) | 4660 (2.08) | 1.18 (0.49-2.84) |
|  | **Nervous system autoimmune diseases** | 20 (2.93) | 4647 (2.08) | 1.50 (0.96-2.36) |
|  | Multiple sclerosis | 16 (3.25) | 4651 (2.08) | 1.78 (1.09-2.90) |
|  | **Gastrointestinal autoimmune diseases** | 66 (1.93) | 4601 (2.08) | 0.96 (0.75-1.24) |
|  | Crohn disease | 22 (2.15) | 4645 (2.08) | 1.08 (0.70-1.66) |
|  | Ulcerative colitis | 24 (1.68) | 4643 (2.08) | 0.86 (0.57-1.30) |
|  | Celiac disease | 24 (1.84) | 4643 (2.08) | 0.89 (0.59-1.35) |
|  | **Other autoimmune diseases** | 20 (2.76) | 4647 (2.08) | 1.36 (0.88-2.11) |
|  | Sarcoidosis | 6 (2.38) | 4661 (2.08) | 1.20 (0.54-2.67) |
|  | IgA nephropathy | 14 (3.23) | 4653 (2.08) | 1.62 (0.96-2.74) |
| **Unnatural cause of death** | **Any AID** | 56 (0.34) | 738 (0.35) | 1.09 (0.82-1.47) |
|  | **Endocrine autoimmune diseases** | 28 (0.36) | 766 (0.35) | 1.36 (0.90-2.05) |
|  | Type 1 diabetes | 11 (0.64) | 783 (0.35) | 1.84 (0.80-4.24) |
|  | Autoimmune thyroiditis | 18 (0.28) | 776 (0.36) | 1.22 (0.76-1.95) |
|  | **Gastrointestinal autoimmune diseases** | 16 (0.47) | 778 (0.35) | 1.43 (0.84-2.42) |
|  | Ulcerative colitis | 7 (0.49) | 787 (0.35) | 1.48 (0.66-3.30) |
|  | Celiac disease | 9 (0.69) | 785 (0.35) | 2.23 (1.11-4.49) |

AID: Autoimmune disease; CI: Confidence interval; HR: Hazard ratio; PY: Person-year.

Only autoimmune diseases with ≥ 5 deaths occurring in the exposed group were included in our analyses.

Analyses were adjusted for offspring’s calendar year of birth, parity, maternal country of birth, age, education, and marital status at the time of birth, maternal smoking in early pregnancy, maternal cardiovascular diseases and diabetes before childbirth.

Supplementary Table S22 Hazard ratios and 95% confidence intervals for mortality in offspring according to maternal autoimmune diseases diagnosed after the child is three years old

| Outcome | Exposure | Events (per 10,000 PYs) | | HR (95% CI) |
| --- | --- | --- | --- | --- |
|  |  | Exposed | Non-exposed |  |
| **All cause of death** | **Any AID** | 597 (3.36) | 5557 (2.69) | 1.19 (1.09-1.30) |
|  | **Endocrine autoimmune diseases** | 300 (3.73) | 5854 (2.71) | 1.29 (1.14-1.46) |
|  | Type 1 diabetes | 39 (6.03) | 6115 (2.74) | 1.68 (1.18-2.38) |
|  | Autoimmune thyroiditis | 264 (3.56) | 5890 (2.72) | 1.25 (1.10-1.42) |
|  | **Inflammatory arthritis** | 69 (3.19) | 6085 (2.74) | 1.16 (0.91-1.48) |
|  | Reactive arthritis | 10 (2.32) | 6144 (2.75) | 0.88 (0.47-1.63) |
|  | Rheumatoid arthritis | 45 (3.38) | 6109 (2.74) | 1.22 (0.90-1.65) |
|  | Ankylosing spondylitis | 15 (3.19) | 6139 (2.74) | 1.06 (0.61-1.82) |
|  | **Vasculitis** | 11 (4.07) | 6143 (2.74) | 1.47 (0.81-2.66) |
|  | Henoch-Schonlein purpura | 5 (3.23) | 6149 (2.74) | 1.13 (0.47-2.73) |
|  | **Connective tissue autoimmune diseases** | 35 (3.76) | 6119 (2.74) | 1.34 (0.95-1.88) |
|  | Systemic lupus erythematosus | 8 (3.24) | 6146 (2.74) | 1.19 (0.60-2.39) |
|  | Sjögren’s disease | 21 (3.85) | 6133 (2.74) | 1.47 (0.96-2.25) |
|  | **Skin autoimmune diseases** | 94 (2.59) | 6060 (2.75) | 0.91 (0.74-1.12) |
|  | Psoriasis vulgaris | 81 (2.56) | 6073 (2.75) | 0.91 (0.73-1.14) |
|  | Vitiligo | 12 (2.78) | 6142 (2.75) | 0.89 (0.48-1.65) |
|  | **Blood autoimmune diseases** | 13 (6.64) | 6141 (2.74) | 2.27 (1.29-4.01) |
|  | Idiopathic thrombocytopenic purpura | 7 (5.36) | 6147 (2.74) | 1.75 (0.79-3.89) |
|  | **Nervous system autoimmune diseases** | 22 (2.40) | 6132 (2.75) | 0.85 (0.55-1.32) |
|  | Multiple sclerosis | 17 (2.50) | 6137 (2.75) | 0.97 (0.60-1.57) |
|  | **Gastrointestinal autoimmune diseases** | 77 (3.04) | 6077 (2.74) | 1.07 (0.85-1.36) |
|  | Crohn disease | 22 (2.96) | 6132 (2.74) | 0.95 (0.60-1.49) |
|  | Ulcerative colitis | 35 (3.25) | 6119 (2.74) | 1.09 (0.77-1.56) |
|  | Celiac disease | 23 (2.93) | 6131 (2.74) | 1.16 (0.77-1.75) |
|  | **Other autoimmune diseases** | 24 (4.60) | 6130 (2.74) | 1.56 (1.03-2.37) |
|  | Sarcoidosis | 12 (3.79) | 6142 (2.74) | 1.31 (0.72-2.36) |
|  | IgA nephropathy | 12 (6.27) | 6142 (2.74) | 2.06 (1.14-3.72) |
| **Natural cause of death** | **Any AID** | 464 (2.61) | 4203 (2.04) | 1.27 (1.15-1.40) |
|  | **Endocrine autoimmune diseases** | 239 (2.97) | 4428 (2.05) | 1.41 (1.23-1.61) |
|  | Type 1 diabetes | 30 (4.63) | 4637 (2.07) | 1.80 (1.21-2.68) |
|  | Autoimmune thyroiditis | 211 (2.84) | 4456 (2.05) | 1.37 (1.19-1.58) |
|  | **Inflammatory arthritis** | 59 (2.73) | 4608 (2.07) | 1.37 (1.05-1.78) |
|  | Reactive arthritis | 9 (2.09) | 4658 (2.08) | 1.07 (0.56-2.06) |
|  | Rheumatoid arthritis | 39 (2.92) | 4628 (2.08) | 1.48 (1.07-2.03) |
|  | Ankylosing spondylitis | 11 (2.33) | 4656 (2.08) | 1.11 (0.60-2.06) |
|  | **Vasculitis** | 6 (2.22) | 4661 (2.08) | 1.09 (0.49-2.43) |
|  | **Connective tissue autoimmune diseases** | 30 (3.22) | 4637 (2.08) | 1.59 (1.10-2.29) |
|  | Systemic lupus erythematosus | 6 (2.43) | 4661 (2.08) | 1.22 (0.55-2.71) |
|  | Sjögren’s disease | 19 (3.48) | 4648 (2.08) | 1.78 (1.14-2.80) |
|  | **Skin autoimmune diseases** | 68 (1.87) | 4599 (2.08) | 0.88 (0.69-1.13) |
|  | Psoriasis vulgaris | 58 (1.83) | 4609 (2.08) | 0.87 (0.66-1.14) |
|  | Vitiligo | 10 (2.32) | 4657 (2.08) | 1.07 (0.56-2.05) |
|  | **Blood autoimmune diseases** | 11 (5.62) | 4656 (2.08) | 2.56 (1.38-4.76) |
|  | Idiopathic thrombocytopenic purpura | 5 (3.83) | 4662 (2.08) | 1.56 (0.59-4.17) |
|  | **Nervous system autoimmune diseases** | 14 (1.53) | 4653 (2.08) | 0.75 (0.43-1.29) |
|  | Multiple sclerosis | 10 (1.47) | 4657 (2.08) | 0.78 (0.42-1.45) |
|  | **Gastrointestinal autoimmune diseases** | 60 (2.37) | 4607 (2.08) | 1.11 (0.85-1.45) |
|  | Crohn disease | 20 (2.69) | 4647 (2.08) | 1.16 (0.72-1.87) |
|  | Ulcerative colitis | 27 (2.51) | 4640 (2.08) | 1.11 (0.73-1.67) |
|  | Celiac disease | 17 (2.17) | 4650 (2.08) | 1.16 (0.72-1.87) |
|  | **Other autoimmune diseases** | 16 (3.06) | 4651 (2.08) | 1.34 (0.79-2.27) |
|  | Sarcoidosis | 7 (2.21) | 4660 (2.08) | 0.97 (0.43-2.15) |
|  | IgA nephropathy | 9 (4.69) | 4658 (2.08) | 2.01 (1.01-4.03) |
| **Unnatural cause of death** | **Any AID** | 71 (0.40) | 723 (0.35) | 0.91 (0.71-1.17) |
|  | **Endocrine autoimmune diseases** | 25 (0.31) | 769 (0.35) | 0.67 (0.44-1.02) |
|  | Autoimmune thyroiditis | 21 (0.28) | 773 (0.36) | 0.63 (0.40-1.00) |
|  | **Inflammatory arthritis** | 8 (0.37) | 786 (0.35) | 0.77 (0.36-1.62) |
|  | **Skin autoimmune diseases** | 20 (0.55) | 774 (0.35) | 1.26 (0.80-1.99) |
|  | Psoriasis vulgaris | 18 (0.57) | 776 (0.35) | 1.36 (0.85-2.17) |
|  | **Gastrointestinal autoimmune diseases** | 11 (0.43) | 783 (0.35) | 1.08 (0.60-1.96) |
|  | Celiac disease | 5 (0.64) | 789 (0.35) | 1.64 (0.68-3.94) |
|  | **Other autoimmune diseases** | 6 (1.14) | 788 (0.35) | 2.79 (1.25-6.23) |

AID: Autoimmune disease; CI: Confidence interval; HR: Hazard ratio; PY: Person-year.

Only autoimmune diseases with ≥ 5 deaths occurring in the exposed group were included in our analyses.

Analyses were adjusted for offspring’s calendar year of birth, parity, maternal country of birth, age, education, and marital status at the time of birth, maternal smoking in early pregnancy, maternal cardiovascular diseases and diabetes before childbirth.

Supplementary Figure S1 Directed acyclic graph illustrating the assumed causal framework for the mediation analysis

Supplementary Figure S2 Cumulative incidence of all-cause mortality in offspring by exposure to maternal autoimmune disease

Supplementary Figure S3 Hazard ratio for all-cause mortality associated with maternal autoimmune diseases over follow-up time estimated using a flexible parametric survival model

The solid line represents the estimated hazard ratio, and the shaded area represents the corresponding 95% confidence interval.

Analysis was adjusted for parity, maternal age, country of birth, education, and marital status at the time of birth.
